# Population structure limits the use of genomic data for predicting phenotypes and managing genetic resources in forest trees

**DOI:** 10.1073/pnas.2425691122

**Published:** 2025-06-25

**Authors:** Gancho T. Slavov, David Macaya-Sanz, Stephen P. DiFazio, Glenn T. Howe

**Affiliations:** ^a^Institute of Biological, Environmental and Rural Sciences, Aberystwyth University, Aberystwyth SY23 3EE, United Kingdom; ^b^Department of Computational and Analytical Sciences, Rothamsted Research, Harpenden AL5 2JQ, United Kingdom; ^c^Department of Biology, West Virginia University, Morgantown, WV 26506-6057; ^d^Departamento de Ecología y Genética Forestal, Instituto de Ciencias Forestales, Consejo Superior de Investigaciones Científicas, Madrid 28040, Spain; ^e^Department of Forest Ecosystems and Society, Oregon State University, Corvallis, OR 97331-5704

**Keywords:** genomic prediction, GWAS, forest trees, climate change, population genetic structure

## Abstract

There is overwhelming evidence that forest trees are locally adapted to climate. Thus, genecological models based on population phenotypes have been used to measure local adaptation, infer genetic maladaptation to climate, and guide assisted migration. However, instead of phenotypes, there is increasing interest in using genomic data for gene resource management. We used whole-genome resequencing and common-garden experiments to understand the genetic architecture of adaptive traits in black cottonwood. We studied the potential of using genome-wide association studies (GWAS) and genomic prediction to detect causal loci, identify climate-adapted phenotypes, and inform gene resource management. We analyzed population structure by partitioning phenotypic and genomic (single-nucleotide polymorphism) variation among 840 genotypes collected from 91 stands along 16 rivers. Most phenotypic variation (60 to 81%) occurred among populations and was strongly associated with climate. Population phenotypes were predicted well using genomic data (e.g., predictive ability *r* > 0.9) but almost as well using climate or geography (*r* > 0.8). In contrast, genomic prediction within populations was poor (*r* < 0.2). We identified many GWAS associations among populations, but most appeared to be spurious based on pooled within-population analyses. Hierarchical partitioning of linkage disequilibrium and haplotype sharing suggested that within-population genomic prediction and GWAS were poor because allele frequencies of causal loci and linked markers differed among populations. Given the urgent need to conserve natural populations and ecosystems, our results suggest that climate variables alone can be used to predict population phenotypes, delineate seed zones and deployment zones, and guide assisted migration.

## Significance

Given climate change, there is an urgent need to conserve natural populations of forest trees and associated ecosystems. In contrast to a growing body of literature on genomic offsets and related approaches, we argue that genomic data will play only an ancillary role in the overall management of forest genetic resources, particularly given the immediate need to respond to climate change and the large number of species that will be affected. Instead, our results suggest that climate variables alone can be used to predict population phenotypes, delineate seed zones and deployment zones, and guide assisted migration.

Forests are key components of global biodiversity and other important ecosystem services, including fuelwood and timber production, regulation of water and air quality, carbon sequestration, climate regulation, and spiritual and recreational experiences (1). However, forests are under pressure from human population growth, conversion of forests to agricultural land, commodity production, wildfire, urbanization, and climate change (2, 3). For example, forest inventories and species distribution models suggest there will be profound shifts in habitats of tree species with climate change (4, 5), likely resulting in maladaptation of locally adapted populations (6–9).

Population-level genetic variation has been studied by measuring phenotypes in common gardens (10–12). These studies established the prevalence of clinal genetic variation along climatic gradients, local adaptation, and greater genetic differentiation for putatively adaptive traits than for neutral genetic markers (i.e., *Q*_ST_ > *F*_ST_) (13–17). Thus, although gene flow is usually extensive in forest trees (18–20), its effects are often outweighed by diversifying selection.

Because climate is a key driver of natural selection, genecological models have been developed to understand the relationships between climate and population-level phenotypes. Growth rate and vegetative bud phenology are typically used as phenotypes because they are consistently associated with climate, genetically correlated with adaptation to cold and drought, and considered surrogates for fitness (9, 14, 21–23). The resulting models have been used to assess the risks of genetic maladaptation from climate change (7, 8) and guide assisted migration (24–28). However, genecological models also have limitations. First, multiple long-term field trials (e.g., >10; 21) are needed to predict field performance accurately, and this is time-consuming and costly. Second, climate-based models do not necessarily account for demographic factors. Third, within-population prediction is limited by the resolution of climate models (29, 30). Overall, genecological models are valuable for inferring deployment areas for breeding populations but contribute little to genetic improvement within populations. Ultimately, genomic information may help overcome some of these limitations (31).

There has been a long-standing interest in using genetic markers instead of phenotypes to manage forest genetic resources. Initially, these studies focused on presumably neutral markers such as allozymes (32, 33) but interest increased dramatically in using genetic markers associated with adaptive traits. Over the past two decades, candidate markers have been identified using association analysis of functional candidate genes (i.e., potential causal loci; 34–36), patterns of gene expression (37, 38), positions relative to mapped QTL in biparental families (16, 39), genotype-environment associations (GEA; 40, 41), and associations with phenotypes via genome-wide association studies (GWAS; 42–46). Despite extensive research on candidate genes, these remain to be validated as causal loci in natural or breeding populations.

GWAS and genomic prediction methods are widely used to study the genetic architectures of complex quantitative traits, meaning the number, locations, and allele frequencies of causal loci plus their additive, dominance, epistatic, and pleiotropic effects (47, 48). Because genetic architecture is often inferred from linked markers, we use “genetic architecture” to refer to the genetic characteristics of causal loci and loci in linkage disequilibrium (LD) with causal loci. The ultimate goal of GWAS is to detect causal loci, identify potential targets for genetic modification, and help predict phenotypes. Although thousands of candidate loci have been identified by GWAS in forest trees, reproducibility has been low, and few have been directly validated (49, 50). Causal loci are difficult to detect because locus effect sizes are small for polygenic traits, GWAS is prone to statistical biases, population structure is often confounded between quantitative traits and neutral markers, and population sample sizes are typically low (e.g., average *N* = 446; 51).

Finally, when GWAS is used among populations, such as in range-wide studies, confounding between phenotypic variation and neutral population structure can lead to many false positive associations (52). Methods exist to mitigate this problem (52) but they are imperfect and may increase false negatives by overcorrecting for population structure. Thus, as described for GEA (53), among-population GWAS suffers from a “Catch-22”—without correcting for population structure, results can be “riddled with false positives,” but causal loci may be missed when corrections are used. Despite the predominance of among-population GWAS in forest trees (49), a substantial proportion of quantitative genetic variation resides within populations (16, 54). This makes within-population GWAS informative and tractable. In humans, for example, it is common to aggregate results from within-population studies across populations using meta-analysis, rather than relying on fundamentally more challenging across-population analyses (55–57).

One way to predict phenotypes is to use many GWAS loci in a single prediction equation (e.g., polygenic score; 57). This is enticing, but the loci must account for a substantial proportion of the phenotypic variation to be valuable, a tall order given that few GWAS associations have been clearly validated in forest trees (49, 50). Alternatively, phenotypes can be predicted using all available markers, assuming most causative loci will be assayed directly or via LD with at least one marker, an approach called genomic selection or genomic prediction (58). This approach, which focuses on prediction rather than identifying causal loci, has been widely used in animal and plant breeding populations (50, 59, 60), humans (61–63), and more rarely, natural populations of forest trees and other plants (64, 65).

Black cottonwood (*Populus trichocarpa*), a fast-growing, riparian tree, is ideal for genomic studies of adaptive traits. It occurs from Baja, California to Alaska (66), inhabits diverse environments, and has well-developed phenotypic and genomic resources (42, 43, 67–70). Because most of the adaptive genetic variation in black cottonwood occurs at the river level (71), we sampled 1,101 clonal genotypes from 23 rivers. Sampling focused on the core of the species range in western Oregon, western Washington, and southwestern British Columbia to avoid the effects of interspecific hybridization and minimize population structure (67) (*SI Appendix, Materials and Methods*). Additionally, to strengthen within-population analyses, we sampled four of these rivers more intensively. We used phenotypic measurements from three replicated field trials and >20 M single-nucleotide polymorphisms (SNPs) from whole-genome resequencing. Then, we used SNPs from a subset of 840 clonal genotypes from 16 rivers to address four questions: 1) How does population genetic structure (i.e., the distribution of genetic variation within versus among populations) differ between SNPs and adaptive trait phenotypes? 2) How do population differences in genetic architecture influence the ability to identify or tag causal loci using GWAS and predict phenotypes within and among populations? 3) How well are phenotypes predicted from SNPs compared to climate and geographic variables? 4) What is the potential for using genomic information from natural populations for gene conservation, breeding, and assisted migration? In contrast to other studies, we combined among-population and within-population analyses to better understand the genetic architecture of adaptive traits in forest trees.

## Results

### Phenotypic Variation in Adaptive Traits was Highly Structured and Strongly Associated with Climate.

**Fig. 1.**
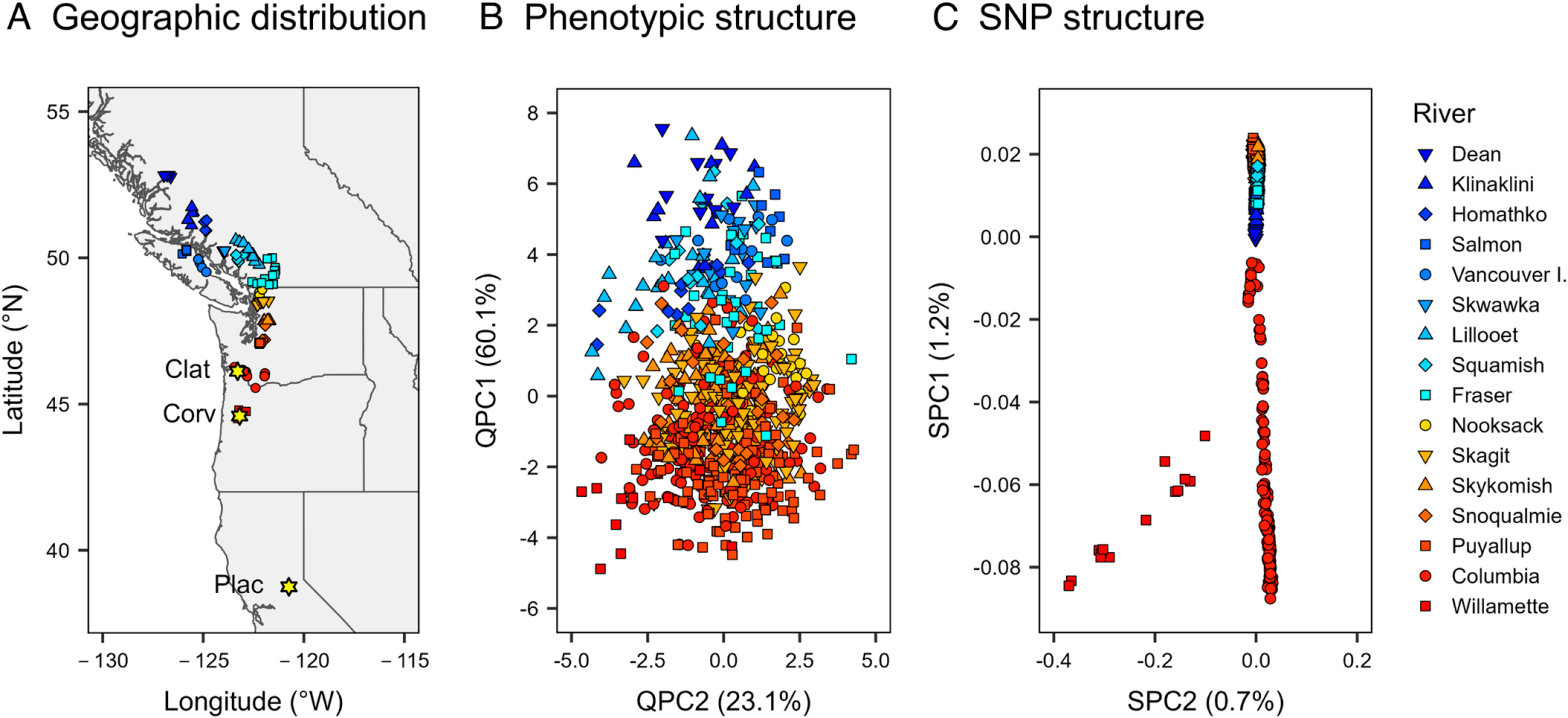
Geographic distribution (*A*), phenotypic population structure (*B*), and SNP population structure (*C*) for 840 *P. trichocarpa* clonal genotypes. (*A*) Source locations are color-coded by river with yellow stars indicating the locations of the three test plantations. (*B*) PC scores (QPC1 and QPC2) from the first two eigenvectors from a principal component analysis (PCA) of BF, BS, and height phenotypes. (*C*) PC scores (SPC1 and SPC2) from the first two eigenvectors from a PCA of SNP markers filtered using “liberal” criteria (*SI Appendix*, Table S4). The values in parentheses are the proportions of total variation accounted for by each PC score based on nine phenotypic variables (*B*) or SNP genotypes (*C*) from 840 clonal genotypes.

**Fig. 2.**
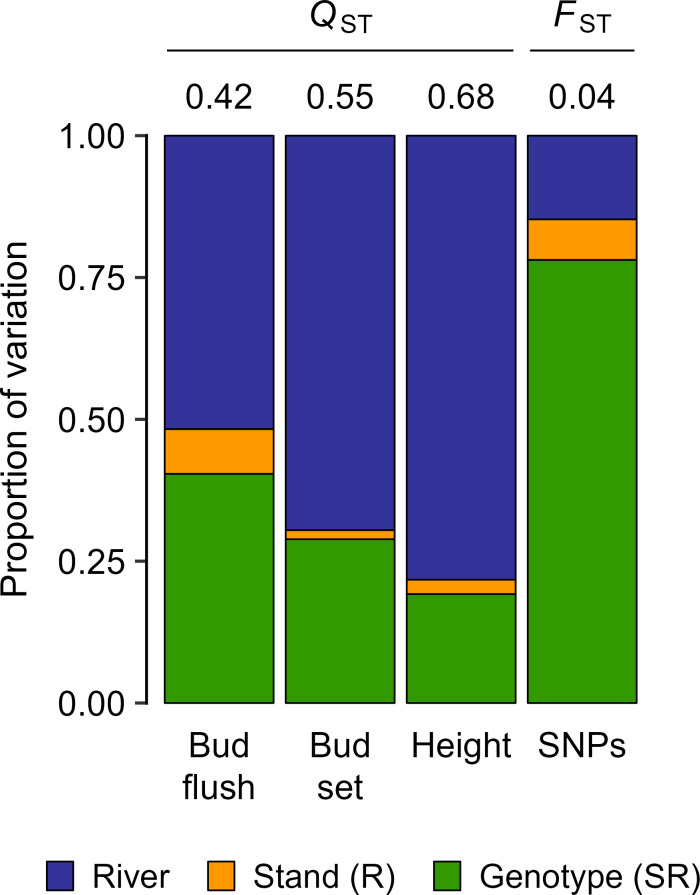
Distributions of genetic variation among rivers [River], stands-within-rivers [Stand (R)], and genotypes-within-stands-and-rivers [Genotype (SR)] for 840 *P. trichocarpa* clonal genotypes. The *y*-axis shows relative proportions of variation based on mixed model analyses of quantitative traits and AMOVA for SNPs. Among-stand *Q*_ST_ values are shown above the bars for three quantitative traits (BF, BS, and height), and the among-stand *F*_ST_ value is shown above the bar for SNPs.

Genetic variation for adaptive traits was highly structured (Fig. 1*B*). For example, the correlation between latitude and the first principal component for quantitative traits (QPC1) was 0.75. Furthermore, differentiation among stands was high (*Q*_ST_ = 0.42 to 0.68, Fig. 2). When genotypic variation for bud flush (BF), bud set (BS), and height was partitioned hierarchically among river, stand-within-river, and genotype-within-stand-and-river levels, more than 50% of the variation occurred at the river level (Fig. 2, first three columns). Finally, based on multivariate regression, phenotypic traits and SNP principal component (SPC) scores were strongly associated with climate (*SI Appendix*, Table S1). Analyses at the river level resulted in similar patterns (*SI Appendix*, Table S2).

### SNP Variation was Moderately Structured and Strongly Associated with Climate.

Although SNP variation had a clear spatial pattern (Fig. 1*C*), the first two principal components explained only 1.9% of the total SNP variation among the 840 clonal genotypes, and the correlation between the first principal component for SNPs (SPC1) and latitude was moderate (*r* = 0.55). In contrast to *Q*_ST_, SNP differentiation was much lower (*F*_ST_ = 0.04, Fig. 2), but varied substantially among SNPs. Based on a sample of 1.1 M SNPs, *F*_ST_ values were as high as 1.00 and the 99th percentile was 0.32. Thus, among all 20.8 M SNPs, there were at least 200 K SNPs with very large *F*_ST_ values. Overall, variation among rivers accounted for 15% of the SNP variation (Fig. 2, fourth blue bar). Finally, variation for the first five SNP PC scores (SPC1-SPC5) was strongly associated with climate (*SI Appendix*, Tables S1 and S2).

### Adaptive Trait Phenotypes Were Predicted Using Geography, Climate, or SNPs.

**Fig. 3.**
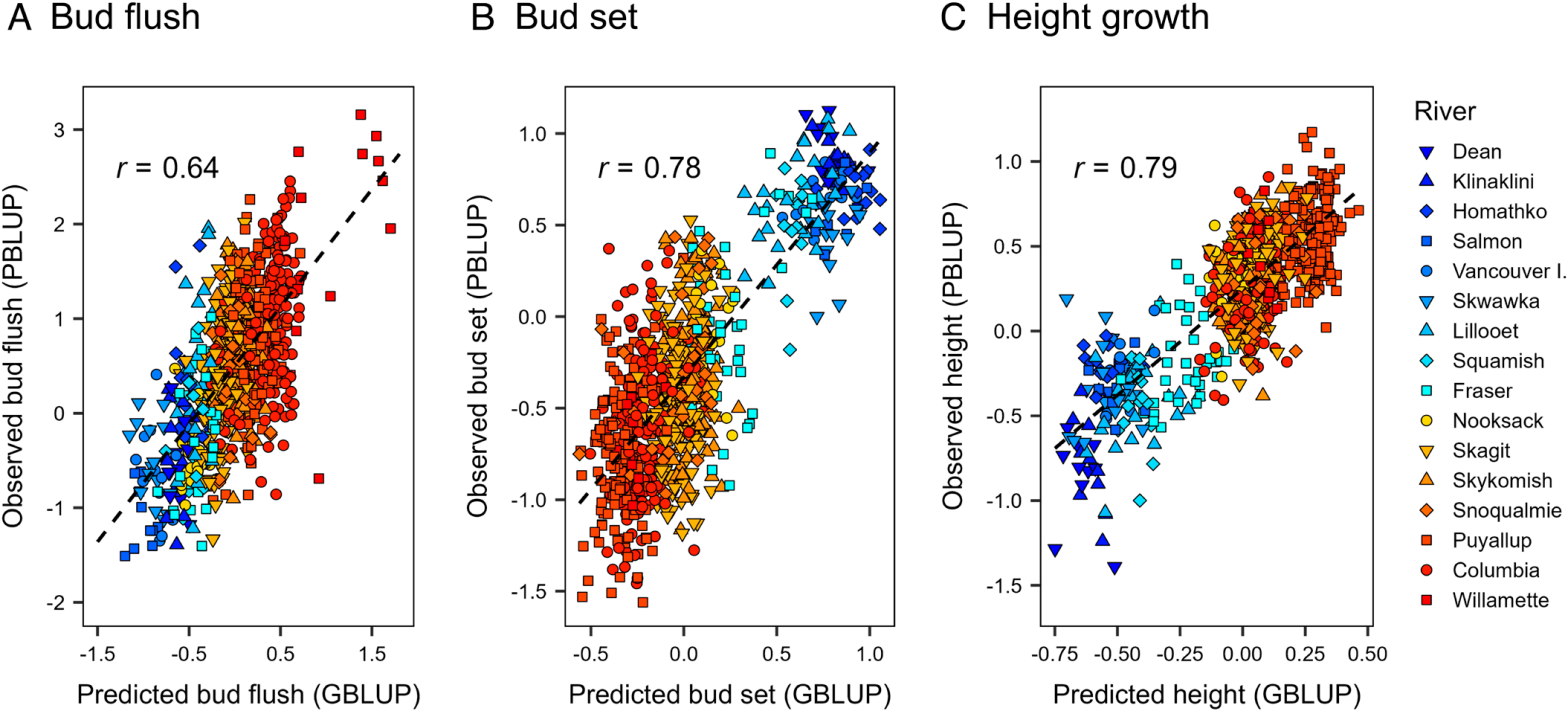
Predicted phenotypic values across all hierarchical levels (genotypes, stands, and rivers) based on field measurements (PBLUP) versus SNPs (GBLUP). Predicted values for BF (*A*), BS (*B*), and height growth (*C*) were based on field measurements of 840 *P. trichocarpa* clonal genotypes or SNP data. GBLUP values are averages of 100 random, 10-fold cross-validations with training population sizes of 756, prediction population sizes of 84, and 20,770,783 SNPs filtered using the liberal criteria (*SI Appendix*, Table S4). The dashed line is the simple linear regression of PBLUP on GBLUP.

**Fig. 4.**
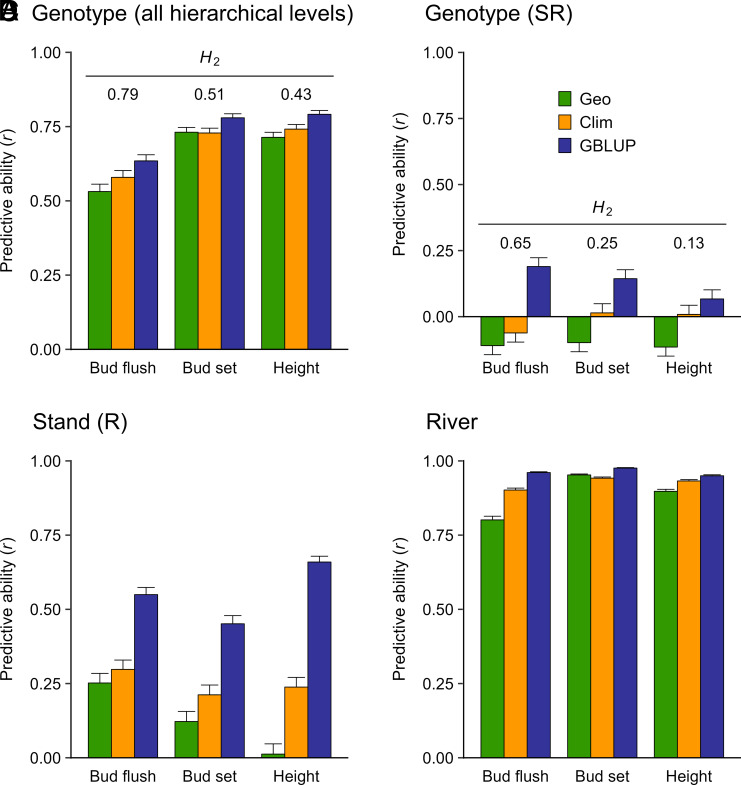
PA for genotypes across all hierarchical levels (*A*), genotypes-within-stands-and-rivers (*B*), stands-within-rivers (*C*), and rivers (*D*). We used ridge regression with three geographic variables (Geo), 21 climatic variables (Clim), and 20,770,783 SNPs (GBLUP) filtered using the liberal criteria (*SI Appendix*, Table S4). Bars are averages from 100 random, 10-fold cross-validations using training population sizes of 756 and prediction population sizes of 84. SE (error bars) were calculated as described in the *SI Appendix, Materials and Methods*. Broad-sense heritabilities (*H^2^*) are shown above the bars in *A* and *B*.

We used phenotypic best linear unbiased predictors (PBLUP) to predict adaptive traits from the measured trees and then compared them to phenotypes predicted from geographic variables, climate variables, or SNPs. These comparisons were evaluated using predictive ability (PA), which is the Pearson correlation between the PBLUP phenotypes versus phenotypes predicted from geographic variables, climate variables, or SNPs. The overall ability to predict phenotypes across stands and rivers was moderate to high (PA > 0.5) using ridge regression with geography, climate, or SNP variables as predictors (Figs. 3 and 4*A* and *SI Appendix*, Table S3). Hereafter, we refer to ridge regression using SNPs or simulated RAD-Seq markers as genomic BLUP (GBLUP). Across traits, GBLUP PAs (0.702 to 0.735) were only modestly higher than PAs based on geography (0.659) or climate (0.683) (*SI Appendix*, Table S3). The absolute advantage of GBLUP was largest for BF. For this trait, GBLUP had a PA of 0.598 to 0.635. In contrast, the PAs were 0.531 based on geography and 0.579 based on climate variables.

### PA was Low after Accounting for Population Structure.

Next, we evaluated PA after rigorously accounting for population structure. By partitioning the PAs into hierarchical levels (Fig. 4 *B*–*D*), three important observations emerged. First, although PAs were moderate to high across the entire population (Fig. 4*A*), none of the models performed well within stands (PA < 0.2, Fig. 4*B*), even though 19 to 40% of the quantitative genetic variation occurred within stands (Fig. 2). Second, GBLUP models based on SNPs were consistently better at predicting stand-level phenotypes than were models based on geography or climate (Fig. 4*C*). Finally, the predictive abilities for river-level phenotypes were high for each model and trait (mean PA = 0.924, range = 0.801 to 0.976; Fig. 4*D*). These results are generally consistent with the hierarchical distribution of genetic variation for phenotypic traits, although within-stand PAs were disproportionately low (Fig. 4*B*) compared to the within-stand genetic variances (Fig. 2, first three green bars).

**Fig. 5.**
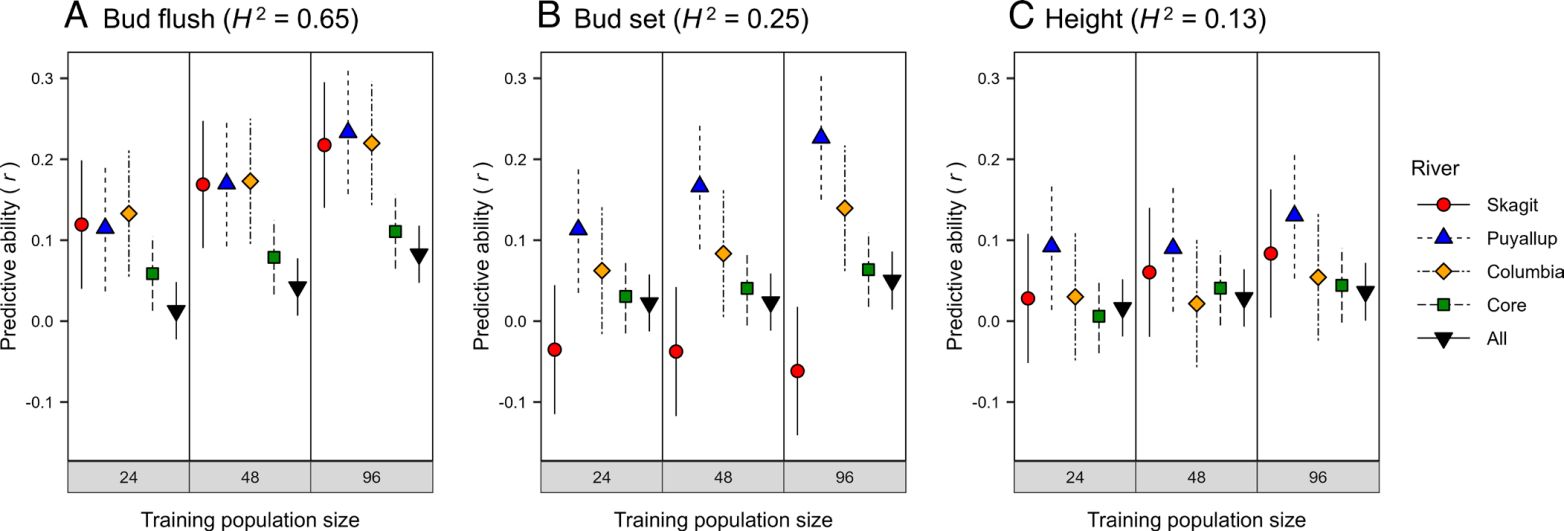
PA for BF (*A*), BS (*B*), and height growth (*C*) as a function of training population size using subsets of genotypes from the Skagit, Puyallup, and Columbia rivers. GBLUP analyses were conducted for genotypes-within-stands-and-rivers [Genotype (SR)] using SNP markers filtered using the liberal criteria (*SI Appendix*, Table S4). PA was calculated using 160 clonal genotypes randomly selected from each of three rivers (Skagit, Puyallup, and Columbia), across all three rivers (Core), or from the entire population (All). Training population sizes were 24, 48, or 96, with a fixed prediction population size of 64 and all 20,770,783 SNPs. Averages were based on 100 replications of each analysis and SE (vertical lines) were calculated as described in the *SI Appendix, Materials and Methods*. Genotype (SR) broad-sense heritabilities (*H^2^*) are shown in parentheses.

The low within-stand PA suggested that genomic prediction was affected by differences in genetic architecture among rivers. To test this, we developed GBLUP models using a subset of data from three well-sampled rivers. Specifically, we compared GBLUP models developed using genotypes sampled within rivers versus across rivers. The PAs of the within-river models (Skagit, Puyallup, and Columbia) were mostly larger or much larger than the PAs of the across-river models (Core and All), irrespective of training population size (Fig. 5). For BF, the PAs for the within-river models were more than twice as large as the PAs across rivers or across the entire study (Fig. 5*A*). We saw similar trends for BS and height growth, but with some anomalies (Fig. 5 *B* and *C*). Because training population size affects PA (Fig. 5 and *SI Appendix*, Fig. S1), PAs may have been higher and more consistent if we had more genotypes per river. For example, PAs increased with increasing training population size in the analyses described above (Fig. 5).

### Few SNP–Phenotype Associations Were Detected after Accounting for Population Structure.

**Fig. 6.**
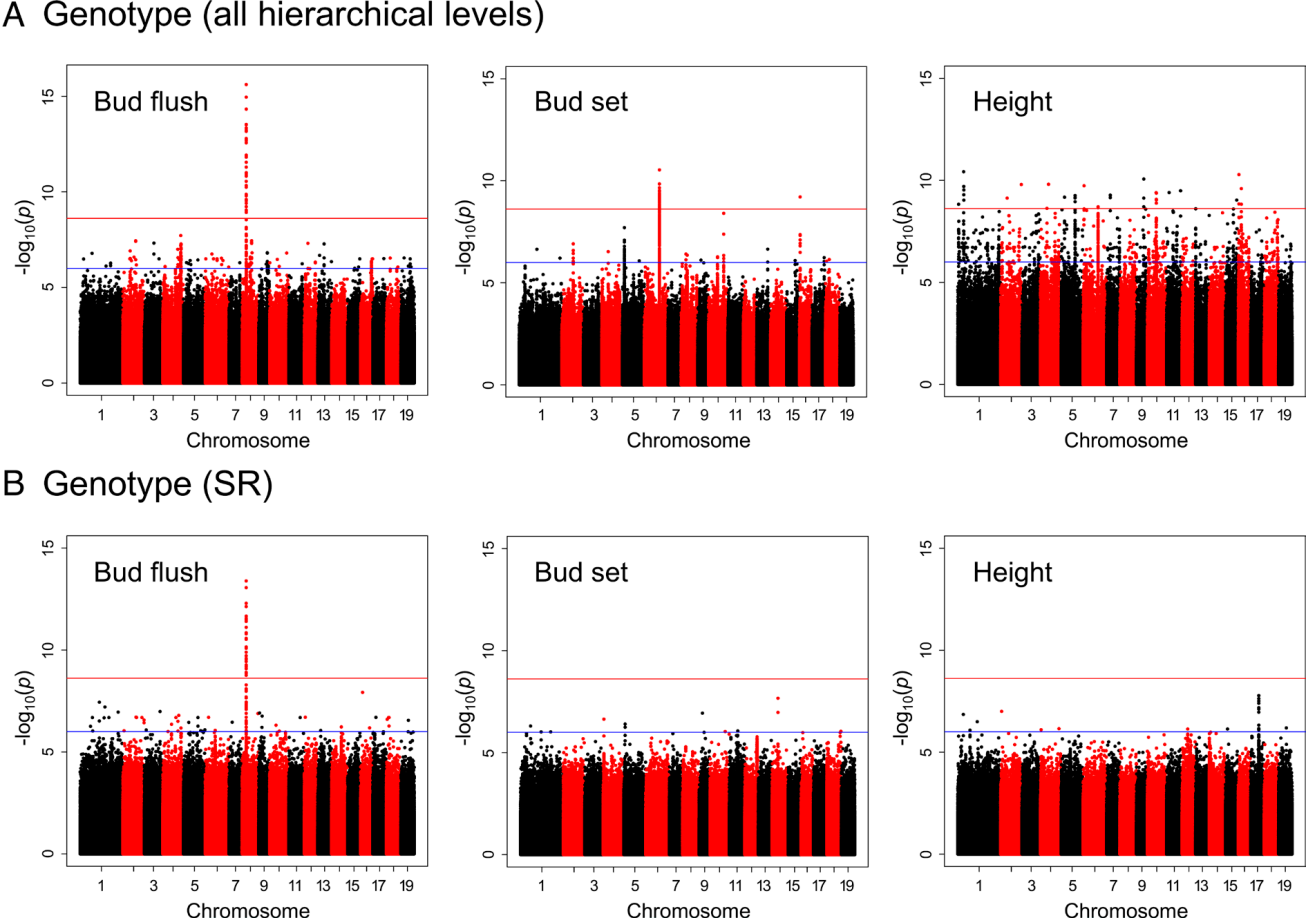
SNP–phenotype associations for BF, BS, and height growth. A GWAS was conducted across all hierarchical levels (*A*) and for genotypes-within-stands-and-rivers (*B*). GWAS was conducted using 20,770,783 SNPs filtered using the liberal criteria (*SI Appendix*, Table S4) and the identity-by-state (IBS) kinship matrix. The blue line indicates a *P*-value of 10^−6^, and the red line indicates a Bonferroni-corrected *P*-value of 2.4 × 10^−9^ (α = 0.05).

To maximize the probability of detecting SNP–phenotype associations, we conducted GWAS using 20.8 M SNPs at two hierarchical levels; within-stands and across-stands-and-rivers. These 20.8 M SNPs were those remaining after using the “liberal” filtering criteria (*SI Appendix*, Table S4). Using analyses designed to account for SNP population structure and cryptic relatedness (72), we detected many associations when we used phenotypes that incorporated variation among genotypes, stands, and rivers (Fig. 6*A* and *SI Appendix,* Fig. S2 *A* and *B*). However, when we conducted the same analyses within stands (i.e., using the genotype-within-stand phenotypes), we detected only one BF association (Fig. 6*B* and *SI Appendix,* Fig. S2 *A* and *B*). Results were similar when we used the first five SNP PC scores (SPC1-SPC5) to correct for population structure, and when we excluded SNPs with minor allele frequency (MAF) < 0.01 (*SI Appendix,* Fig. S2 *C*–*F*). In contrast, there was little difference between the two types of analyses (i.e., across all hierarchical levels versus among genotypes-within-stands-and-rivers) when we analyzed a less structured subset of genotypes from the Skagit, Puyallup, and Columbia Rivers (*SI Appendix,* Fig. S2 *G* and *H*). In both cases, only the single BF association was detected.

### Within-Stand Genomic Prediction and GWAS Were Probably Limited by Population Differences in Genetic Architecture.

We hypothesized that population differences in genetic architecture were partly responsible for the low PAs and few SNP associations within stands. To test this, we examined two important components of genetic architecture—allele frequencies at causal loci and LD. Population differences in other components of genetic architecture, such as allele effect sizes and epistasis, may have also contributed (56) but were not studied because much larger samples would be needed.

First, we considered population differences in allele frequencies at causal loci (e.g., QTN or quantitative trait nucleotides). Allele frequencies affect the percentage of variation explained by a locus (PVE) and, thus, the ability to detect SNP–phenotypic associations using GWAS or predict phenotypes using GBLUP. Because we tested 20.8 M SNPs (one SNP every 20 bp, *SI Appendix,* Table S4), our analyses likely included most of the common adaptive trait QTN (i.e., excluding QTN with MAF < 0.003). As described above, allele frequency differences among rivers were substantial. Many pairwise *F*_ST_ values among rivers were close to 0.10 or greater (*SI Appendix,* Fig. S3*A*) and allele frequency differences among rivers averaged 0.05 (SD = 0.06). Finally, differences in allele frequencies were structured—17% of SNPs (i.e., >3 M SNPs) had allele frequencies that were correlated with latitude at the river level (i.e., *P* < 0.05). Thus, for the causative loci alone, among-river differences in MAF and PVE are probably substantial––leading to the poor success of GWAS and genomic prediction using pooled within-population analyses.

**Fig. 7.**
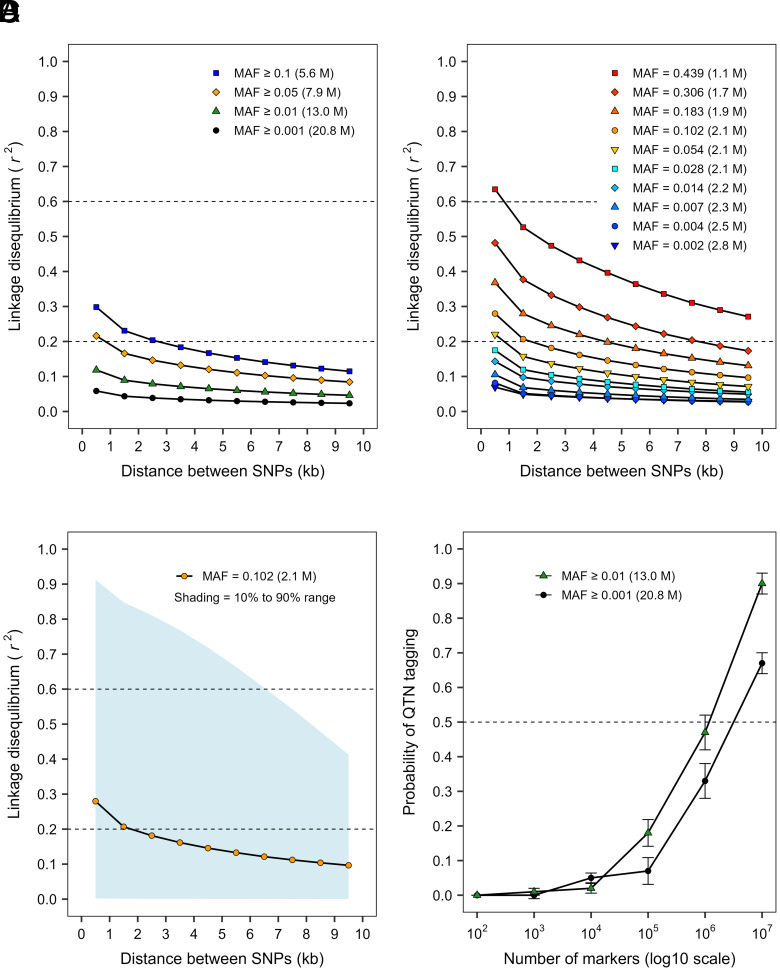
LD and probability of tagging causative loci using different MAF thresholds. LD was calculated using different MAF filtering criteria (*A*), by MAF bin (*B*), and for a bin with MAF ranging between 0.071 and 0.132 (midpoint = 0.102) (*C*). MAF bin ranges (*SI Appendix*, Table S4) are represented by their midpoints, and LD was calculated as the average *r*^2^ for pairs of SNPs in each 1-kb distance class. The probability of tagging a hypothetical QTN was calculated using different numbers of randomly selected SNPs (*D*). Tagging was defined as the presence of at least one SNP in LD (*r*^2^ ≥ 0.6) with the QTN within 10 kb. Averages and SE (error bars) were based on 100 random samples. Numbers of SNPs are shown in parentheses.

Second, we studied population differences in LD, which may result from differences in demographic history, allele frequencies, or linkage phases between loci. The extent of LD (i.e., average *r*^2^ > 0.2) varied roughly threefold among rivers (6 to 18 kb) and was on average 29% higher within rivers than across rivers (*SI Appendix*, Fig. S4). The relationship between LD and physical distance varied substantially by MAF (Fig. 7*A*). Thus, we also quantified LD in MAF bins chosen to ensure all pairs of loci in a bin could have an *r*^2^ of at least 0.5 (*SI Appendix*, Table S5). In these analyses, LD was near zero for bins containing rare SNPs (MAF < 0.01). In contrast, for common SNPs (MAF ≥ 0.10), LD extended from 2 to 3 to over 10 kb (Fig. 7*B*). Because *r*^2^ values were highly variable, even within MAF bins (Fig. 7*C*), we estimated the probability that a causative polymorphism (e.g., QTN) would be tagged (*r*^2^ ≥ 0.6) by at least one SNP within 10 kb (Fig. 7*D*). This probability was sensitive to MAF filtering and the number of SNPs used to tag the QTN. Overall, more than 1 M SNPs would be needed to tag a QTN with a probability of 0.5. Many more SNPs would be required to tag QTNs with even greater confidence, particularly if the QTN allele is rare (Fig. 7*D*). Thus, allele frequency differences among stands and rivers resulted in corresponding differences in LD, which likely affected the within-stand GBLUP and GWAS analyses.

**Fig. 8.**
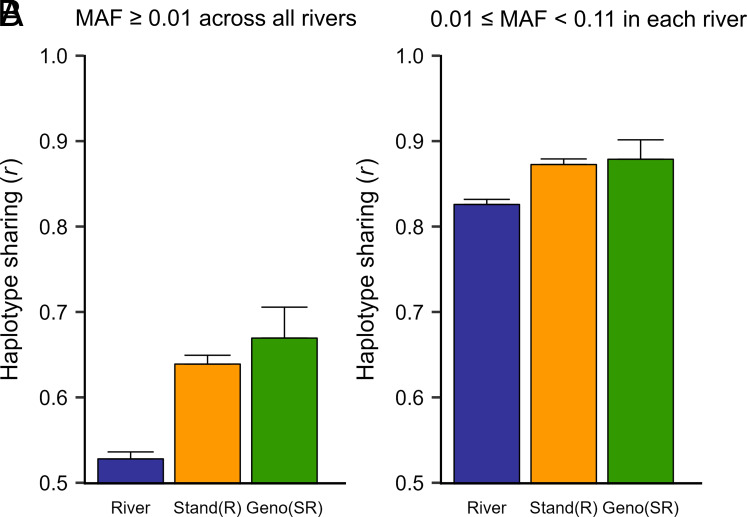
Linkage phase consistency (haplotype sharing) among genotypes-within-stands-and-rivers [Geno (SR)], stands-within-rivers [Stand (R)], and rivers (River). (*A*) Analyses were based on 1,082,633 SNPs with MAF ≥ 0.01 separated by at least 300 bp and haplotype sharing was calculated for all pairs of SNPs located within 10 kb of each other. (*B*) Analyses were based on 1,002 SNPs with 0.01 ≤ MAF < 0.11 in each of the 16 rivers and haplotype sharing was calculated for all pairs of SNPs located within 1 Mb of each other. SE (error bars) were calculated as described in the *SI Appendix, Materials and Methods*.

Differences in linkage phase between QTN and linked markers, such as those resulting from different population histories, will also contribute to population variation in LD. To evaluate this contribution, we calculated haplotype sharing, a measure of linkage phase and allele frequency consistency among individuals, either within or among populations. Haplotype sharing was 17 to 21% lower for individuals from different rivers compared to individuals from the same stand or from different stands within rivers (Fig. 8*A*). Patterns of haplotype sharing for pairs of rivers strongly resembled those for allele frequency differentiation (*SI Appendix,* Fig. S3, *r* = −0.87, *P* < 10^−6^ from a Mantel test), suggesting that allele frequency differences were largely responsible for the reduced haplotype sharing among rivers. Indeed, haplotype sharing was much higher when analyses were limited to SNPs that had similar allele frequencies in each river, but the difference among hierarchical levels was still detectable (Fig. 8*B*).

### Delineation of Seed Zones.

**Fig. 9.**
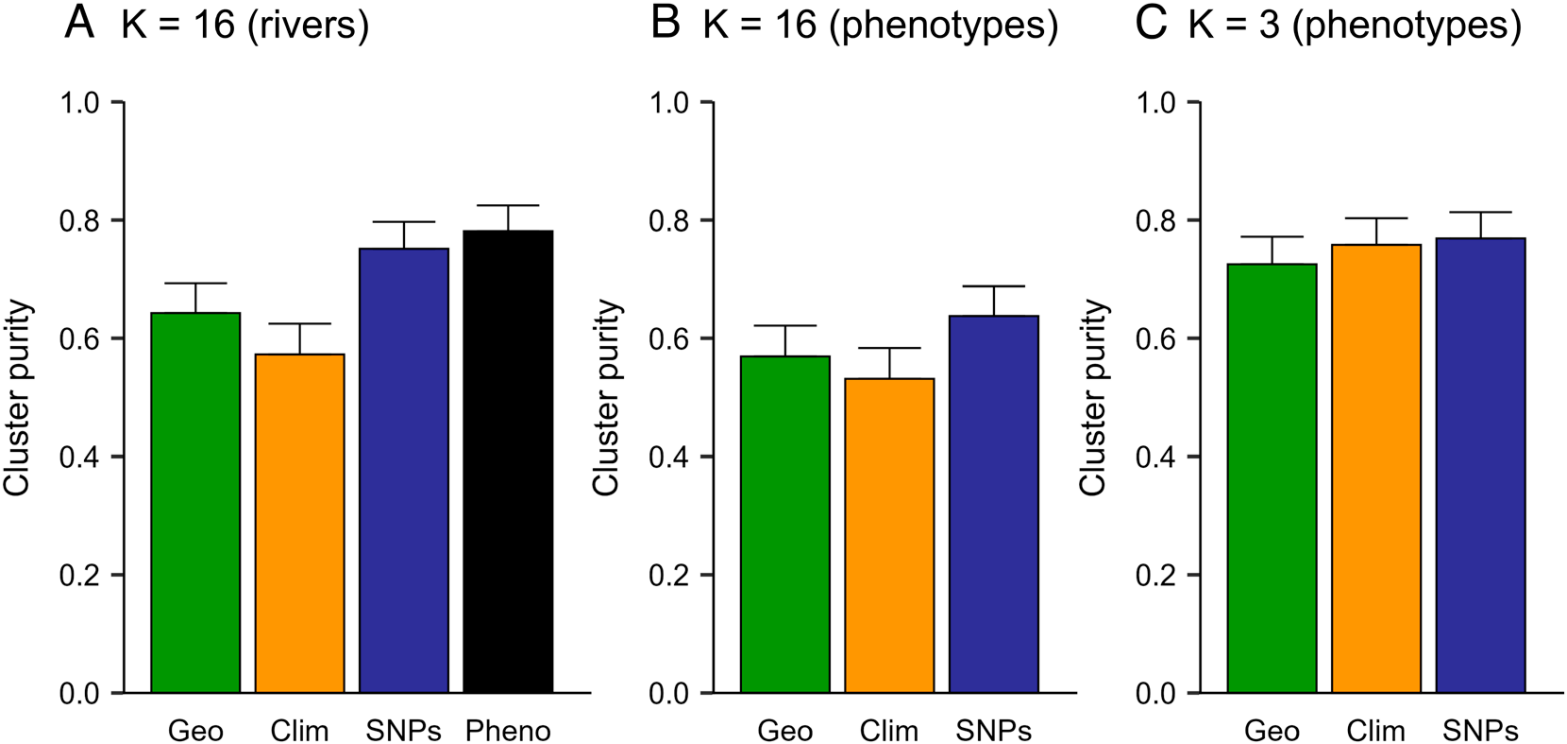
Delineation of stand-level seed zones using geographic (Geo), climate (Clim), SNP, or phenotypic data (Pheno) for 840 *P. trichocarpa* clonal genotypes sampled from 91 stands in 16 rivers. Different numbers (K) of true seed zones were assumed to correspond to rivers (*A*), or seed zones were defined using k-means clustering of phenotypes (*B* and *C*). The success of reconstructing true seed zones based on different types of data was evaluated using cluster purity, which is the proportion of stands that were both in the same true seed zone and in the same reconstructed seed zone. Averages and SE (error bars) were based on 100 replications of each analysis and calculated as described in the *SI Appendix, Materials and Methods*.

To illustrate the practical implications of our results, we compared the ability of different types of data (i.e., phenotypic, geographic, climate, and SNPs) to reconstruct seed deployment zones delineated using different criteria (Fig. 9). Seed zones are geographic areas of genetic and environmental homogeneity used to guide deployment (e.g., planting) of tree genotypes (73). For natural populations, we assume that genotypes collected within a seed zone can be deployed within the same zone without risking maladaptation. When the “true” seed zones were assumed to correspond to the 16 rivers (Fig. 9*A*), phenotypic data were best for reconstructing these zones, although SNPs were only slightly less accurate (cluster purity of 0.782 versus 0.752). Purity is a 0 to 1 measure of cluster quality, or the extent to which a clustering method recovers known classes. The cluster purities of geographic and climate data were substantially lower for this scenario (0.643 and 0.573). However, when true seed zones were delineated based on phenotypic data, reconstructions based on geographic, climate, and SNP data had more similar cluster purities (Fig. 9 *B* and *C*).

## Discussion

Our results highlight the challenges of using genomic information to understand the genetics of complex quantitative traits in natural populations. Because climate is a primary driver of local adaptation (74), we focused on climate adaptation traits, or simply “adaptive traits.” These traits have been used for guiding tree gene conservation, breeding, and assisted migration (73, 75–77). We studied height growth and vegetative bud phenology using GWAS and genomic prediction, two widely used approaches in forest trees (49, 50, 78).

GWAS has been used in natural populations—with the goal of identifying causal loci (42, 79, 80), enhancing tree breeding (81, 82) or informing assisted migration (83). Nonetheless, many studies are compromised by population structure, the inclusion of closely related individuals, low marker coverage, or small population sizes (i.e., low power) (49, 84). These limitations may lead to misidentification of causal loci and other misinterpretations. In contrast, genomic prediction works best in populations of closely related trees and where identification of causal loci is not an explicit goal. Thus, genomic prediction has been mostly used to select desirable genotypes in breeding populations of forest trees (reviewed in ref. 78).

We used GWAS and genomic prediction to understand climate adaptation traits in natural populations of black cottonwood. In particular, we partitioned genetic variation into hierarchical levels to understand how population structure affects inferences about complex quantitative traits. Our results highlight the challenges of using GWAS and genomic prediction across populations with different genetic architectures. Finally, compared to genomic information, population-level phenotypes were predicted nearly as well by climate alone.

### Genomic Prediction and GWAS Were Highly Sensitive to Population Genetic Structure.

We showed that phenotypic variation in adaptive traits was highly structured and strongly associated with climate, which is consistent with other studies of black cottonwood and other wide-ranging tree species (7, 8, 17, 42, 44, 69, 71, 85–89). In contrast, SNP variation was moderately structured but clearly associated with climate. Other population genomic studies found similar evidence for SNP population structure, but patterns of variation were typically much weaker than for adaptive traits, both in *Populus* (42, 88–90) and other trees (16, 17). In our study, the difference between phenotypes (average *Q*_ST_ = 0.55) and SNPs (overall *F*_ST_ = 0.04) was pronounced but not surprising, given that most SNPs are probably selectively neutral.

When we conducted analyses across all hierarchical levels (i.e., rivers, stands, and genotypes), we detected one association with BF, two associations with BS, and over a dozen associations with height growth. However, when we accounted for population structure using SNP PCs (42, 52) and by conducting within-population analyses, most associations disappeared. This suggests they were false positives caused by population structure—but could indicate the presence of causal loci strongly differentiated among populations. Thus, other lines of evidence (e.g., based on networks of gene coexpression, comethylation, or association with metabolites) would be needed to infer the biological functions of these loci (91, 92). Ultimately, gene editing provides a powerful tool for functional validation of genes implicated by GWAS (49).

Although the number of GWAS hits declined after correcting for population structure, a single BF association remained significant. This association was found even after excluding rare alleles (i.e., MAF < 0.01) and analyzing a subset of genotypes from three rivers in the core of the species range. This BF association involved 30 common SNPs (MAF ~ 0.10, *P*-value < 2.4 × 10^−9^) in strong LD, spanning a region of nearly 60 kb. The same association was reported by Evans et al. (42), but McKown et al. (44) found no BF associations in this region in a study of black cottonwoods sampled mostly from British Columbia. This difference highlights the influence of genetic architecture on the ability to detect causal loci using GWAS.

In GWAS, uncorrected population structure will likely lead to misidentification of causal loci—and the numbers of false positives can become very large as more SNPs are analyzed. For example, among the 20.8 M SNPs we analyzed, ~3.5 M had allele frequencies that were significantly correlated with latitude (uncorrected *P* < 0.05). In these cases, even rigorous accounting for population structure may fail (52). Thus, because adaptive traits tend to correlate with population structure, only associations that are also detected within populations should be considered robust.

Likewise, our ability to predict phenotypes using SNP markers mostly resulted from population structure—PAs were high at the river level (0.950 to 0.976), moderate at the stand-within-river level (0.451 to 0.659), and low for genotypes within stands (0.067 to 0.190). In contrast, to the other hierarchical levels, within-stand prediction likely resulted from linkage between SNPs and causal loci or the ability of SNPs to estimate relatedness among trees.

What are implications for gene resource management? First, there is little to be gained by using SNPs to predict phenotypes for rivers or stands. At the river level, prediction was almost as good as using climate variables alone (Fig. 4*D*). At the stand level, SNPs were better predictors than climate variables (Fig. 4*C*), but only 2 to 8% of the genetic variation occurred at that level (Fig. 2). If phenotypes are available from field tests, within-stand prediction could be used to expand existing breeding populations—but PAs are very low and wild genotypes are rarely infused after the first generation of breeding. Also, genomic prediction would need to be weighed against directly comparing new field selections with advanced-generation genotypes in field tests where family or clonal heritabilities can be very high.

Although we focused on height growth and phenology traits, population structure will make it difficult to understand the genetic basis of other climate adaptation traits as well. For example, drought tolerance is a complex quantitative trait with pronounced population structure associated with climate (93, 94). However, plant biochemical traits (95) or other traits with little population structure should be more amenable to GWAS and genomic prediction. Finally, GWAS should work well for traits controlled by one or a few genes, such as major gene resistance to white pine blister rust disease (96).

### Population-Level Phenotypes Can Be Predicted Using SNPs or Climate Variables.

There has been much interest in using genomic information to infer maladaptation to future climates and guide assisted migration (83, 97, 98). Thus, a variety of statistical approaches have been developed to predict maladaptation from genomic data (e.g., genomic offsets, 83) that are analogous to earlier approaches using phenotypes (7, 8, 99). Using phenotypes measured in the field, we demonstrated that climate adaptation traits can be predicted using SNPs but were predicted nearly as well using climate variables alone. Furthermore, there were few differences between seed zones delineated using phenotypes, SNPs, or climate variables (Fig. 9). Genomic offset experiments suggest that SNP-based genomic offsets can be used to predict population phenotypes better than climate or geographic variables alone, but not consistently (100, 101). These conclusions are generally consistent with our results but the value of SNP versus climate information seemed to be less pronounced in our study, at least at the river level.

As we found using genomic prediction, the performance of genomic offsets seems to rely on population structure—random markers performed as well as known causal markers in simulations (102) and as well as candidate loci in empirical studies (100, 101), but see ref. 103. In addition to being surrogates for phenotypic population structure, SNPs may enhance prediction by reducing error in climate variables. Predictions from climate interpolation models are not without error, particularly in remote and mountainous regions and when low-resolution climate data are used (e.g., 1 × 1 km, 100, 101)—and adding SNP data may counteract these errors. If so, climate-only models might be improved by increasing the accuracy of climate data; perhaps by establishing networks of ‘micro’ weather stations (30). Because this would improve assisted migration for all species, it might be a wiser use of resources compared to developing new genomic resources for many individual species.

SNPs might also improve predictions by accounting for phenotypic relationships among populations unrelated to climate—e.g., those resulting from demographic processes such as colonization, migration, or secondary contact. In any case, based on our results and genomic offset studies, it is unlikely that the predictive power of genomic offsets comes from information derived from causal loci. On the other hand, using SNPs to guide assisted migration has two potential pitfalls. First, neutral population structure may follow different spatial patterns compared to phenotype-climate associations, leading to poor prediction of maladaptation. Second, because the acquisition of SNP data will probably delay assisted migration for most species, it might be more pragmatic to use climate-only models instead. Simulations suggest that a priori selection of climate variables improves climate-only models (102). Thus, because phenotype-climate associations are reasonably well understood across species (16, 28, 104), we argue that important climate variables can be reasonably selected a priori and used to guide assisted migration in the absence of SNP data.

When phenotypic and genomic data are unavailable, provisional conservation and assisted migration decisions can be made using climate alone. Novel climates, which are good candidates for gene conservation, can be identified by clustering stands using multivariate climate distance functions (105) and an analogous approach can be used to delineate seed zones. Likewise, climate distances among locations can be used to practice assisted migration (106, 107). Source populations, which are assumed to be well adapted to recent historical climates, may be deployed to locations where the projected future climates are similar (i.e., match). A climate match is one with a climate distance less than or equal to the “climate distance threshold” (CDT), which is the climate distance beyond which tree performance is expected to be unacceptable. Although it is best to use provenance tests to infer CDTs, Shalev et al. (107) discuss alternative approaches. Using a multivariate climate distance function, the most robust matches are those that fall within the CDT using multiple climate projections.

### Why Were PAs So Low and Why Did We Find Few GWAS SNPs within Populations?

Given the large number of SNPs we used, why was it difficult to detect associations and predict phenotypes within populations (i.e., after rigorously accounting for population structure)? Based on our results and interpretation of the relevant literature, we offer four main explanations: 1) complex quantitative traits are controlled by many genes with small effects, 2) frequencies of causative polymorphisms differ among populations, 3) LD is mostly low, particularly for rare alleles, and 4) frequencies of marker alleles and LD differ among populations.

Evidence suggests most traits in forest trees are controlled by many loci with small effects (50, 51), and studies of outcrossing plants, livestock, and humans lead to similar conclusions (108–110). These factors have three important effects for complex quantitative traits. First, very large sample sizes will be needed to detect most small-effect loci, probably many more than have been used or perhaps are even feasible (110). Second, low-powered experiments are likely to report many spurious associations (49, 50). Finaly, thousands of GWAS loci may be needed to explain most of the genetic variation in quantitative traits. The challenges in understanding the genetic basis of human height provide a cautionary tale. Recent success at explaining most of the variation in human height (e.g., > 50%) required millions of study participants and more than 12 K independent GWAS loci (i.e., SNP associations) (111).

Causal loci are difficult to detect when allele frequencies differ among populations (56). Because we used 20.8 M markers with an average spacing of about 20 nt across the genome, we probably genotyped most of the causal QTN, yet detected few GWAS loci. The power to detect a causal locus (*c*) depends on sample size (*N*) and the proportion of phenotypic variance explained by the causal locus (PVE_*c*_), where PVE*_c_* is a function of allele effects (e.g., standardized regression coefficient, β) and MAF: ${\text{PVE}}_{c}=2\cdot \beta^{2}\cdot \mathrm{MAF}\;(1-\mathrm{MAF})$ (110). Thus, important contributors to GWAS power are *MAF*, number of contributing loci (reflected in the standardized regression coefficients), and experimental *N*. When MAF varies across populations, GWAS power also varies, which contributes to poor reproducibility. Additionally, it is unlikely that all causal loci can be assayed using SNPs alone because phenotypic variation also arises from other types of genomic variation (112). Finally, population differences in LD (i.e., *r*^2^) reduce power even further when linked markers (*m*) are used to detect causal loci. In this case, ${\text{PVE}}_{m}=r^{2}\cdot {\text{PVE}}_{c}$ (110).

Generally, low LD in forest trees has been attributed to an outcrossing mating system, large effective population size, weak selection, and little population structure for most loci (113, 114). However, more recent studies revealed exceptions (115), and generally indicate that LD is higher and more variable across the genome than previously thought (67, 116, 117). In our study, LD for common SNPs (i.e., MAF > 0.1) decayed below 0.2 within 1 to 3 kb on average, and extended well beyond 10 kb for more than 10% of SNP pairs (Fig. 7*C*). On the other hand, LD was near zero for rare SNPs (MAF < 0.01, Fig. 7 *A* and *B*). Mostly low LD and small locus effect sizes make it difficult to identify causal loci using linked markers. Furthermore, GWAS power is particularly low when allele frequencies differ between markers and causal loci (118). That is, differences in allele frequencies can make causal loci “invisible” to most nearby markers. In our study, an LD > 0.6 seemed necessary to detect the single BF locus. Using 0.6 as the LD cut-off, more than 1 M SNPs with MAFs > 0.01 would be needed to have a 50% chance of tagging a causal locus (Fig. 7*D*). Overall, our ability to detect the BF locus seemed to rely on a high MAF for the causal locus (i.e., > 0.05), high heritability for the phenotypic trait (0.79), and large locus effect size (i.e., PVE ~ 5%).

Population differences in linkage phase may also obscure species-wide associations using linked markers—SNPs associated with positive phenotypes in one population may be associated with negative phenotypes in another. This may have been a contributing factor in our study because haplotype sharing was greatest within stands and lowest among rivers (Fig. 8*A*). However, we showed that the reduced haplotype sharing among rivers was mostly due to differences in allele frequencies (Fig. 8*B*). Thus, differences in linkage phase are probably not the main reason for low GWAS power and PA.

Overall, while other differences in genetic architecture (e.g., allele substitution effects or epistasis) may also be contributing, we hypothesize that the main limiting factors for GWAS and genomic prediction are allele frequency differences in causal and marker loci among populations (56, 57). Across-population analyses lead to incorrect inferences about the causal relationships between SNPs and phenotypes, whereas pooled within-population analyses have low power to detect GWAS loci or predict phenotypes.

### Implications.

We show that across-population GWAS and genomic prediction are strongly influenced by population structure, rather than the causal relationships between SNP loci and adaptive traits. Thus, across-population analyses promote incorrect inferences about causal loci. Instead, analyses of single populations or the use of pooled within-population analyses should lead to more robust conclusions. The drawback of using a single population is that causal loci may be missed because they are not segregating. The drawback of using pooled within-population analyses is that power is compromised by differences in genetic architecture among populations. In any case, to detect most SNP–trait associations and predict phenotypes accurately, population sample sizes in the tens to hundreds of thousands will probably be needed. Obviously, experiments of this size will be infeasible for most forest tree species, even for a single population. Furthermore, based on human studies, substantially larger experiments may be needed (111). Thus, we conclude that GWAS analyses are unlikely to detect most of the causal loci, explain a substantial proportion of trait heritability, or contribute meaningfully to traditional tree breeding, gene conservation, or assisted migration. GWAS can almost certainly be used to detect some of the causal loci, but perturbing expression in transgenic plants or gene editing may ultimately be required to validate causal loci (49). Likewise, the success of within-population genomic prediction will improve as sample sizes become larger, but predictive abilities in most natural populations will always be constrained by the low relatedness among trees. Compared to the predictive abilities of progeny tests alone, it is questionable if genotyping and other costs needed to use genomic prediction in natural populations will be justified for adaptive trait breeding or assisted migration.

Despite the challenges, substantial research has been devoted to identifying or tagging causal loci for practical applications such as assisted migration. In contrast, our results demonstrate the power of using neutral loci or climate variables to predict population-level phenotypes, at least for species with local adaptation to climate. Black cottonwood differs from other tree species in having a mostly riparian distribution, substantial amounts of vegetative reproduction, and interspecific hybridization. Nonetheless, our results are expected to be relevant to many temperate zone tree species, including conifers. First, we designed our collections to sample populations that had a common demographic history unaffected by recent introgression from other *Populus* species (i.e., figures 1-1 and 1-2 in ref. 119). Second, we focused on the portion of the range where black cottonwood has optimal growth, high levels of phenotypic variation, and weak interpopulation differentiation for neutral markers (66, 67, 86, 120). This resulted in levels of population differentiation similar to many other tree species for quantitative traits (*Q*_ST_) and neutral genetic markers (*F*_ST_) (17). Our results are less relevant for tropical and subtropical species that have little climate-based population structure, but may be relevant for species exposed to geographic patterns in seasonal drought (121). Thus, our conclusions mostly apply to locally adapted plant and animal species for which assisted migration is considered (25).

We hypothesized that SNPs improve climate-based prediction of population phenotypes by helping to characterize population structure, particularly when inappropriate climate variables are used or when the climate variables have error. Given the urgent need to conserve natural populations and ecosystems, our results suggest that climate variables alone can be used to predict population phenotypes, delineate seed zones and deployment zones, and guide assisted migration.

## Materials and Methods

### Plant Materials and Test Plantations.

In the winter of 2008, we obtained or collected stem cuttings from 1,101 *P. trichocarpa* genotypes, representing a large portion of the latitudinal range of the species (Fig. 1*A*; 67). In April and May of 2009, we established rooted cuttings in three test plantations spanning the south-central portion of the black cottonwood range west of the Cascade Mountains (Fig. 1*A* and *SI Appendix, Materials and Methods*).

### Phenotypic Measurements and Analysis.

Between 2009 and 2013, we measured height growth and two phenological traits, vegetative BF and BS, by visually classifying the phenological state of each tree using six-stage scoring scales (*SI Appendix*, Fig. S6). For each plantation and year, we chose measurement dates to maximize the phenotypic variation in BF and BS. In addition, we measured the current and previous year heights of the main stem as the distance from the groundline to the apical bud or to the most recent bud scale scars (i.e., position of last year’s apical bud). For data analyses of height growth (HT), we averaged height growth for the 2010 to 2012 growing seasons. Similarly, when multiple BF and BS measurements were available, we first identified the measurement with the highest heritability for a given year (*SI Appendix, Materials and Methods*) and then averaged measurements across years. Finally, we used mixed linear models to estimate variance components, heritabilities, genetic correlations, and random effects (i.e., BLUPs) at the river (R), stand-within-river [S(R)], and genotype-within-stand-and-river [G(SR)] levels (*SI Appendix, Materials and Methods*). Thus, this approach allowed us to partition genetic variance and calculate “phenotypes” at three hierarchical levels (i.e., river, stand, and genotype), as well as across all levels (G).

### SNP Data.

We obtained data for 28,342,758 biallelic SNPs (https://cbi.ornl.gov/gwas-dataset/) from 970 *P. trichocarpa* individuals (clonal genotypes) and then removed 130 individuals from this dataset for the final analyses. We excluded individuals with a mean sequencing depth <7, eliminated close relatives using an approach similar to that of Evans et al. (42), and then excluded 42 other individuals for other reasons (*SI Appendix, Materials and Methods*). The remaining 840 clonal genotypes represented 91 stands in 16 rivers (Fig. 1). For the final analyses, we used VCFtools v. 0.1.14 (122) and PLINK v.1.90b4.4 (123) to filter SNPs based on “strict” and “liberal” criteria, and then simulated a set of 51,820 “RAD-Seq” markers (*SI Appendix*, Table S4 and *Materials and Methods*).

### SNP Population Structure and Allele Frequency Differences.

We calculated individual-tree PC scores using the liberally filtered SNP set and the SMARTPCA software package (v. 13050; 124). For this analysis, we selected a subset of nonsingleton SNPs separated by at least 300 bp (*vcftools --thin* 300), and then removed one SNP from each pair of loci linked at *r^2^* ≥ 0.8 to avoid artifacts caused by large blocks of tightly linked markers (124, 125). Bivariate plots of PCs were used to reveal population structure at the river level. We also used SMARTPCA to calculate pairwise estimates of *F*_ST_ at the river level based on Hudson’s estimator, which is robust to the effects of rare-allele SNPs (126). Finally, we used the same SNPs and the HIERFSTAT package in R (127) to estimate SNP variance components and hierarchical *F*-statistics at the river, stand, and genotype levels. This analysis was designed to match our analyses of phenotypic data (see above, *SI Appendix, Materials and Methods*).

To quantify allele frequency differences among rivers, we first calculated the allele frequencies of all liberally filtered SNPs (*plink --freq --family*) in each river. Then, we calculated pairwise allele frequency differences among rivers and correlations between the river-level allele frequencies and latitude using R (128).

### GBLUP and GWAS.

We used the *kin.blup* function of the rrBLUP R package (129) to predict phenotypes based on SNP markers (i.e., GBLUP approach; 58, 130). Genomic relationship matrices (GRMs) were calculated using the *kin.blup* function of rrBLUP or the --*make-grm-alg 1* option of GCTA (131). We also used a subset of analyses to test various Bayesian approaches implemented in the BGLR R package (132), and the results were essentially the same. The phenotypes for BF, BS, and HT were the random effects for three hierarchical levels, G(SR), S(R), and R, as well as the combined effects across all levels (G), using random effects from Model 2 (*SI Appendix, Materials and Methods*). We tested the effect of training population size (*N_t_*) and numbers of SNP markers for some analyses and evaluated the performance of GBLUP using PA, which is the Pearson product-moment correlation coefficient between the input phenotypes and the phenotypes predicted from the SNP data (*SI Appendix, Materials and Methods*) (59, 133). Finally, we compared the GBLUP approach described above (i.e., based on the GRM alone) to models that also included the first five PC scores of the genomic relationship matrix as fixed-effect covariates (63).

We performed GWAS analyses on the G(SR) and G phenotypes using the methods described in refs. 42 and 133. Briefly, we used the EMMAX software to implement the Efficient Mixed Model Association Expedited approach (72). Models for all GWAS analyses included the identity-by-state kinship matrix to control for cryptic relatedness and population structure. A subset of analyses also included the first five PCs from the SMARTPCA analyses described above as fixed-effect covariates (52).

### LD and Haplotype Sharing.

Across all hierarchical levels, we calculated *r*^2^ for each pair of SNPs located within 10 kb of each other using different MAF cut-offs or bins (*SI Appendix*, Table S5 and *Materials and Methods*). We used these data to estimate the probability of tagging a randomly assigned (hypothetical) QTN. This probability was calculated as the proportion of times at least one SNP within 10 kb had *r*^2^ ≥ 0.6 with the hypothetical QTN. For the within-river analyses, we used the same approach but equalized sample sizes within rivers using random subsampling (*SI Appendix, Materials and Methods*).

To quantify linkage phase consistency among rivers, stands-within-rivers, and genotypes-within-stands-and-rivers, we calculated haplotype sharing (134–136) at each of these levels (*SI Appendix, Materials and Methods*).

### Geographic and Climatic Random Forest and Ridge Regression Analyses.

We used rrBLUP and random cross-validations to compare the predictive abilities of SNPs versus those obtained using geographic and climatic variables. The geographic variables consisted of latitude, longitude, and elevation, whereas the climate variables consisted of 21 temperature and precipitation-related variables from ClimateNA v5.21 (29).

We used rrBLUP to be consistent with the GBLUP analyses described above. We also used lasso regression to evaluate the relative importance of the geographic and climatic variables. Because lasso regression involves variable selection, it is useful for interpreting the relative importance of the regression predictors. The details of these analyses are described in the *SI Appendix, Materials and Methods*.

### Delineation of Seed Zones.

We evaluated the performance of phenotypes, SNPs, climate variables, and geographic variables for delineating seed zones. Although *Populus* species are typically propagated clonally, the term “seed zone” is often used to denote native populations of forest trees with sufficient genetic homogeneity to be treated as a single population for reforestation purposes. We used three methods to delineate the true or target seed zones and then compared these to “reconstructed” zones delineated using phenotypes or ridge regression predictions (i.e., for SNPs, climate variables, and geographic variables). We delineated true zones by 1) assuming they corresponded to the 16 rivers, 2) using K-means clustering to delineate 16 zones based on the phenotypes, and 3) using K-means clustering to delineate three zones based on the phenotypes. To compare the true versus reconstructed zone allocations, we calculated cluster purity (137), which is the proportion of stands in each reconstructed seed zone that were also in the same true zone (*SI Appendix, Materials and Methods*).

## Supplementary Material

Appendix 01 (PDF)

Dataset S01 (XLSX)

## Data Availability

Genome resequencing data have been deposited in CBI data repository (https://cbi.ornl.gov/gwas-dataset/). Previously published data were used for this work (42). All other data are included in the article and/or supporting information.
