# Supplementary material for: Population structure limits the use of genomic data for predicting phenotypes and managing genetic resources in forest trees
Source: Proc Natl Acad Sci U S A. 2025 Jun 25;122(26):e2425691122. doi: 10.1073/pnas.2425691122 (PMC12232740; doi:10.1073/pnas.2425691122)
Supplement: Supplementary file 1 — Appendix 01 (PDF) [file pnas.2425691122.sapp.pdf]

PNAS

Appendix SI

Supporting Information for

Population structure limits the use of genomic data for predicting phenotypes and managing genetic resources in forest trees

Gancho T. Slavov, David Macaya-Sanz, Stephen P. DiFazio, Glenn T. Howe

Corresponding author: Gancho T. Slavov
Email: gas38@aber.ac.uk

This PDF file includes:

Supporting Results
Tables S1 to S5
Figures S1 to S6
Supporting Materials and Methods
SI References

Other supporting materials for this manuscript include the following:

Dataset S1

Supporting Results

Predictive ability increased with training population size and number of markers

As expected, GBLUP PAs increased with the size of the training population (*N*) and when the number of markers (*M*) was increased from 10 to 10,000,000 (Fig. S1). These trends differed between analyses using phenotypic variation across all hierarchical levels (Fig. S1A, B) and those for genotypes within stands (Fig. S1C, D). Predictive abilities tended to increase with *N* at a higher rate in the within-stand analyses (Fig. S1C) than in analyses conducted across all hierarchical levels (Fig. S1A). Furthermore, whereas increasing *M* beyond 100,000 had little effect on PA in analyses across all hierarchical levels (Fig. S1B), a plateau was reached at a higher *M* (1,000,000) in analyses within stands (Fig. S1D). In contrast, SNP filtering had little effect on GBLUP PAs. For example, the mean PA across traits was the same (0.735) using SNPs filtered with strict versus liberal filtering criteria (Tables S3, S4). Furthermore, PA was only slightly lower (0.702) using a much smaller set of 51,820 simulated RAD-Seq markers (Tables S3, S4).

Genotype × environment interactions had little effect on predictive abilities

We examined the magnitude of genotype-by-environment (G×E) interactions using (1) variance components from analyses of phenotypic data and (2) among-plantation genetic correlations for each trait. GxE variance accounted for only 9-20% of the total genetic variation, and genetic correlations ranged from 0.80 to 0.95 for the same trait measured in different plantations (Fig. S5A). Consequently, there was only a small decrease in PA when GBLUP models of phenotypic data from one test plantation were trained on phenotypic data from another (Fig. S5B).

Supporting Tables and Figures

Table S1. Stand-level prediction of adaptive traits and SNP PC scores using random forest (RF) and ridge regression (RR). Dependent variables were stand-level BLUPs for quantitative traits (bud flush, BF; bud set, BS; and height growth, HT) and stand-level PC scores for single-nucleotide polymorphisms (SNPs) (SPC1-SPC5). Predictive abilities (PA)* were calculated using RF and RR and four sets of independent variables: (1) subsets of climate variables selected using RF at the stand level, (2) all 21 climate variables, (3) geographic variables (latitude, longitude, and elevation), and (4) all climate and geographic variables.† SE is the mean standard error of PA across all models and analyses (RF and RR) for each trait. Each SE was based on 100 model runs.

		RF-selected climate variables		All climate variables		Geographic variables		All climate and geographic variables		
Trait	Climate variables selected using random forest at the stand-level	RF	RR	RF	RR	RF	RR	RF	RR	SE
Quantitative traits		***Predictive ability****								
BF	Eref + EXT + DD<0 + TD + MCMT + FFP	0.851	0.791	0.831	0.793	0.899	0.746	0.877	0.808	0.0010
BS	Eref + DD<0 + TD	0.919	0.866	0.900	0.903	0.976	0.950	0.976	0.965	0.0004
HT	Eref + DD<0 + TD	0.910	0.839	0.885	0.889	0.951	0.880	0.949	0.922	0.0005
SNP PC scores										
SPC1	Eref + DD<0 + MCMT + TD + PAS + MSP + SHM	0.920	0.654	0.900	0.730	0.931	0.739	0.931	0.818	0.0012
SPC2	Eref	0.818	-0.089	0.595	0.107	0.822	-0.028	0.728	0.205	0.0140
SPC3	Eref + TD + CMD + EMT + MSP + bFFP	0.760	0.656	0.729	0.689	0.913	0.666	0.891	0.756	0.0015
SPC4	MSP + SHM + CMD + DD>5 + Eref + DD<18 + DD<0 + EMT + TD	0.849	0.733	0.826	0.774	0.956	0.791	0.938	0.873	0.0008
SPC5	Eref	0.534	-0.032	0.334	-0.011	0.469	0.011	0.373	-0.010	0.0087

* Predictive ability is the correlation between the input dependent values (BLUPs or PC scores) and the values predicted using RF or RR using 10-fold cross-validation.

† Annual climate variables are the day of the year on which the frost-free period begins (bFFP), Hargreaves climatic moisture deficit (CMD), degree-days below 0°C (DD<0), degree-days below 18°C (DD<18), degree-days above 5°C (DD>5), extreme minimum temperature over 30 years (EMT), Hargreaves reference evaporation (Eref), extreme maximum temperature over 30 years (EXT), frost-free period (FFP), mean coldest month temperature (MCMT), May to September precipitation (MSP), mean warmest month temperature (MWMT), summer heat-moisture index (SHM = MWMT/(MSP/1000)), precipitation as snow (PAS), and temperature difference between MWMT and MCMT (TD)(1).

Table S2. River-level prediction of adaptive traits and SNP PC scores using random forest (RF) and ridge regression (RR). Dependent variables were river-level BLUPS for quantitative traits (bud flush, BF; bud set, BS; and height growth, HT) and river-level PC scores for single-nucleotide polymorphisms (SNPs) (SPC1-SPC5). Predictive abilities (PA)* were calculated using RF and RR and four sets of independent variables: (1) subsets of climate variables selected using RF at the stand-level, (2) all 21 climate variables, (3) geographic variables (latitude, longitude, and elevation), and (4) all climate and geographic variables.† SE is the mean standard error of PA across all models and analyses (RF and RR) for each trait. Each SE was based on 100 model runs.

		RF-selected climate variables		All climate variables		Geographic variables		All climate and geographic variables		
Trait	Climate variables selected using random forest at the stand-level	RF	RR	RF	RR	RF	RR	RF	RR	SE
Quantitative traits					***Predictive ability****					
BF	Eref + EXT + DD<0 + TD + MCMT + FFP	0.753	0.865	0.440	0.338	0.467	0.100	0.410	0.347	0.0071
BS	Eref + DD<0 + TD	0.848	0.888	0.853	0.775	0.850	0.908	0.874	0.804	0.0028
HT	Eref + DD<0 + TD	0.830	0.853	0.902	0.850	0.900	0.875	0.908	0.869	0.0033
SNP PC scores										
SPC1	Eref + DD<0 + MCMT + TD + PAS + MSP + SHM	0.423	-0.230	0.401	-0.177	0.456	-0.008	0.404	-0.181	0.0145
SPC2	Eref	-0.830	-0.693	-0.421	-0.703	-0.449	-0.651	-0.434	-0.702	0.0123
SPC3	Eref + TD + CMD + EMT + MSP + bFFP	0.553	0.370	0.515	0.486	0.592	0.396	0.523	0.498	0.0037
SPC4	MSP + SHM + CMD + DD>5 + Eref + DD<18 + DD<0 + EMT + TD	0.751	0.577	0.773	0.674	0.823	0.580	0.788	0.686	0.0023
SPC5	Eref	0.111	-0.555	0.020	-0.570	-0.322	-0.555	0.005	-0.586	0.0083

* Predictive ability is the correlation between the input dependent values (BLUPS or PC scores) and the values predicted using RF or RR using 8-fold cross-validation.

† Annual climate variables are the day of the year on which the frost-free period begins (bFFP), Hargreaves climatic moisture deficit (CMD), degree-days below 0°C (DD<0), degree-days below 18°C (DD<18), degree-days above 5°C (DD>5), extreme minimum temperature over 30 years (EMT), Hargreaves reference evaporation (Eref), extreme maximum temperature over 30 years (EXT), frost-free period (FFP), mean coldest month temperature (MCMT), May to September precipitation (MSP), mean warmest month temperature (MWMT), summer heat-moisture index (SHM = MWMT/(MSP/1000)), precipitation as snow (PAS), and temperature difference between MWMT and MCMT (TD)(1).

Table S3. Predictive abilities for phenotypic values across all hierarchical levels (genotypes, stands, and rivers) using GBLUP (SNP predictor variables) or ridge regression (geographic or climate variables) for 840 *Populus trichocarpa* genotypes. SNP (single-nucleotide polymorphism) markers were filtered using strict, liberal, or 'Rad-Seq' marker (Rad) criteria (Table S4). All predictive abilities are based on 100 random, 10-fold cross-validations with a training population size of 756 and a prediction population size of 84. Numbers in parentheses are the number of variables used (in column headings) or standard errors (in the table body; see Supporting Materials and Methods).

		Predictive ability (PA) based on N = 840 genotypes				
		GBLUP*			Ridge regression (RR)†	
Trait‡	H^{2}§	Strict SNP markers (3,175,407)	Liberal SNP markers (20,770,783)	Rad markers (51,820)	Geographic variables (3)	Climate variables (21)
BF	0.794	0.631 (0.027)	0.635 (0.027)	0.598 (0.028)	0.531 (0.029)	0.579 (0.029)
BS	0.512	0.784 (0.022)	0.780 (0.022)	0.740 (0.024)	0.731 (0.024)	0.728 (0.024)
HT	0.427	0.789 (0.021)	0.791 (0.021)	0.767 (0.023)	0.714 (0.025)	0.742 (0.025)
***Average*¶**	**0.578**	**0.735**	**0.735**	**0.702**	**0.659**	**0.683**

*GBLUP is the predictive ability using 3,175,407 markers filtered using 'strict' criteria, 20,770,783 markers filtered using 'liberal' criteria, or 51,820 markers filtered using 'RAD-Seq' criteria (Table S4).

† Ridge regression is the predictive ability using ridge regression with 3 geographic variables or 21 climate variables.

‡ Trait is the BLUP prediction for bud flush (BF), bud set (BS), or height growth (HT).

§ H^2 is the broad-sense heritability.

¶ Average is the overall average across traits.

Table S4. Filtering criteria for single-nucleotide polymorphism (SNP) data from 840 *Populus trichocarpa* genotypes.

**Filtering criteria***	***Strict***	***Liberal***	***RAD-Seq***
Min depth	8	NA	NA
GATK genotype quality	20	NA	NA
Missing (%)	10	NA	NA
Minor allele count	17†	2	2
Minor allele frequency	0.01	0.0012‡	0.0012‡
No. of alleles	2	2	2
**Statistics§**	***Strict***	***Liberal***	***RAD-Seq***
No. of loci	3,175,407	20,770,783	51,820
Max gap (bp)	702,736	56,822	2,033,113
No. >10 kb gaps	2,112	355	7,605
Ave gap (bp)	124	20	7,576
Med gap (bp)	29	9	36
Ave missing (%)	6.6	11.0	1.4
Ave MAF	0.15	0.08	0.09
Med MAF	0.10	0.02	0.03

* Filtering criteria include *Min depth*: minimum number of reads per genotype; *GATK genotype quality*: Phred-scaled confidence that the genotype assignment is correct (i.e. difference between the log10-transformed likelihoods of the second most likely and the most likely genotype); *Missing (%)*: maximum percentage of missing genotype data allowed for a given locus; *Minor allele count*: minimum number of copies of the minor allele among all genotypes; *Minor allele frequency*: minimum frequency of the minor allele among all genotypes; and *No. of alleles*: number of SNP alleles detected; and NA: not applicable.

† Calculated from minor allele frequency.

‡ Calculated from minor allele count.

§ *Statistics include No. of loci*: number of SNP loci that passed all filtering criteria; *Max gap (bp)*: largest distance between adjacent SNPs; *No. >10 kb gaps*: number of distances between adjacent SNPs exceeding 10 kb; *Ave gap (bp)*: average distance between adjacent SNPs; *Med gap (bp)*: median distance between adjacent SNPs; *Ave missing (%)*: average percentage of missing genotype data; *Ave MAF*: average minor allele frequency; *Med MAF*: median minor allele frequency.

Table S5. Minor allele frequency (MAF) bins[*] used for analyses of linkage disequilibrium.

Bin	MAF_{min}	MAF_{max}	$MAF_{midpoint}$	N_{SNPs}	Ave. gap (bp)	Median gap (bp)
1	0.0012	0.0024	0.0018	2,790,927	146	62
2	0.0024	0.0047	0.0036	2,482,036	166	65
3	0.0047	0.0094	0.0071	2,335,579	177	65
4	0.0094	0.0187	0.0141	2,204,361	187	68
5	0.0187	0.0367	0.0277	2,096,945	195	69
6	0.0367	0.0709	0.0538	2,123,088	192	68
7	0.0709	0.1324	0.1016	2,075,169	195	67
8	0.1324	0.2338	0.1831	1,870,724	216	68
9	0.2338	0.3790	0.3064	1,664,173	242	71
10	0.3790	0.5000	0.4395	1,127,781	355	83

[*] MAF_{min} and MAF_{max} were the lower and upper bounds of bins calculated to assure that all pairs of loci in a bin could have an r^2 with a theoretical maximum of at least 0.5 (see Wray (2)); $MAF_{midpoint}$ were the midpoints of each bin [(MAF_{min} + MAF_{max})/2]; N_{SNPs} were the number of SNPs in each bin; Ave. gap (bp) were the average gap sizes (i.e., distance between adjacent SNPs) and Median gap (bp) were the median gap sizes for each bin.

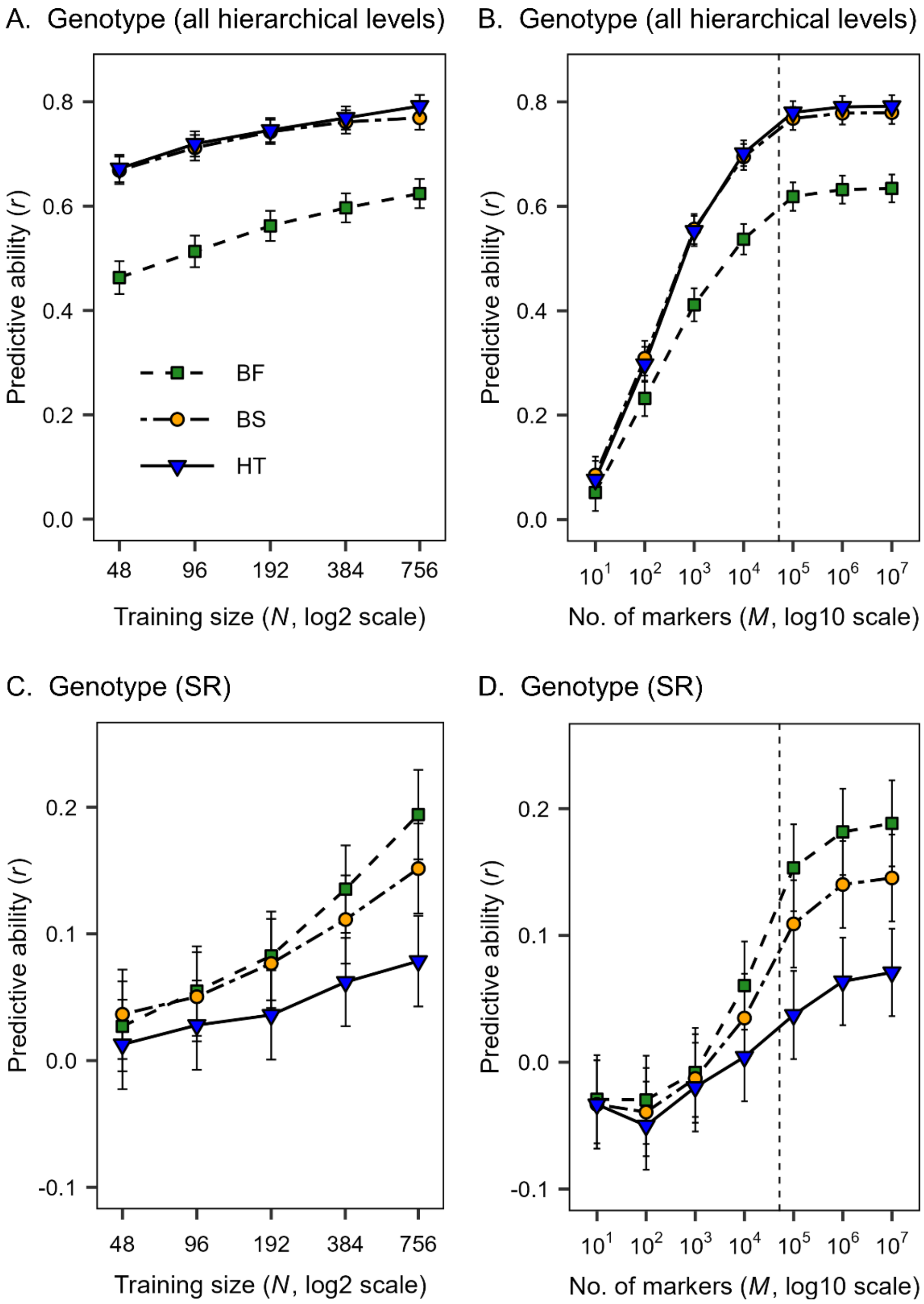

Figure S1. Predictive ability versus training population size (A, C) and number of markers (B, D) using 840 clonal genotypes. GBLUP analyses were conducted across all hierarchical levels (A, B) and for genotypes-within-stands-and-rivers [Genotype (SR)] (C, D) using single-nucleotide polymorphism (SNP) markers filtered using the 'liberal' criteria (Table S4). In A and C, training population sizes ranged from 48 to 756, with a fixed prediction population size of 84, and 20,770,783 SNPs. In B and D, the number of SNPs ranged from 10 to 10,000,000, using a training population size of 756 and a prediction population size of 84. Averages were based on 100 replications and standard errors (error bars) were calculated as described in the Supporting Materials and Methods. The dashed line shows the number of simulated RAD-Seq markers we used in some analyses.

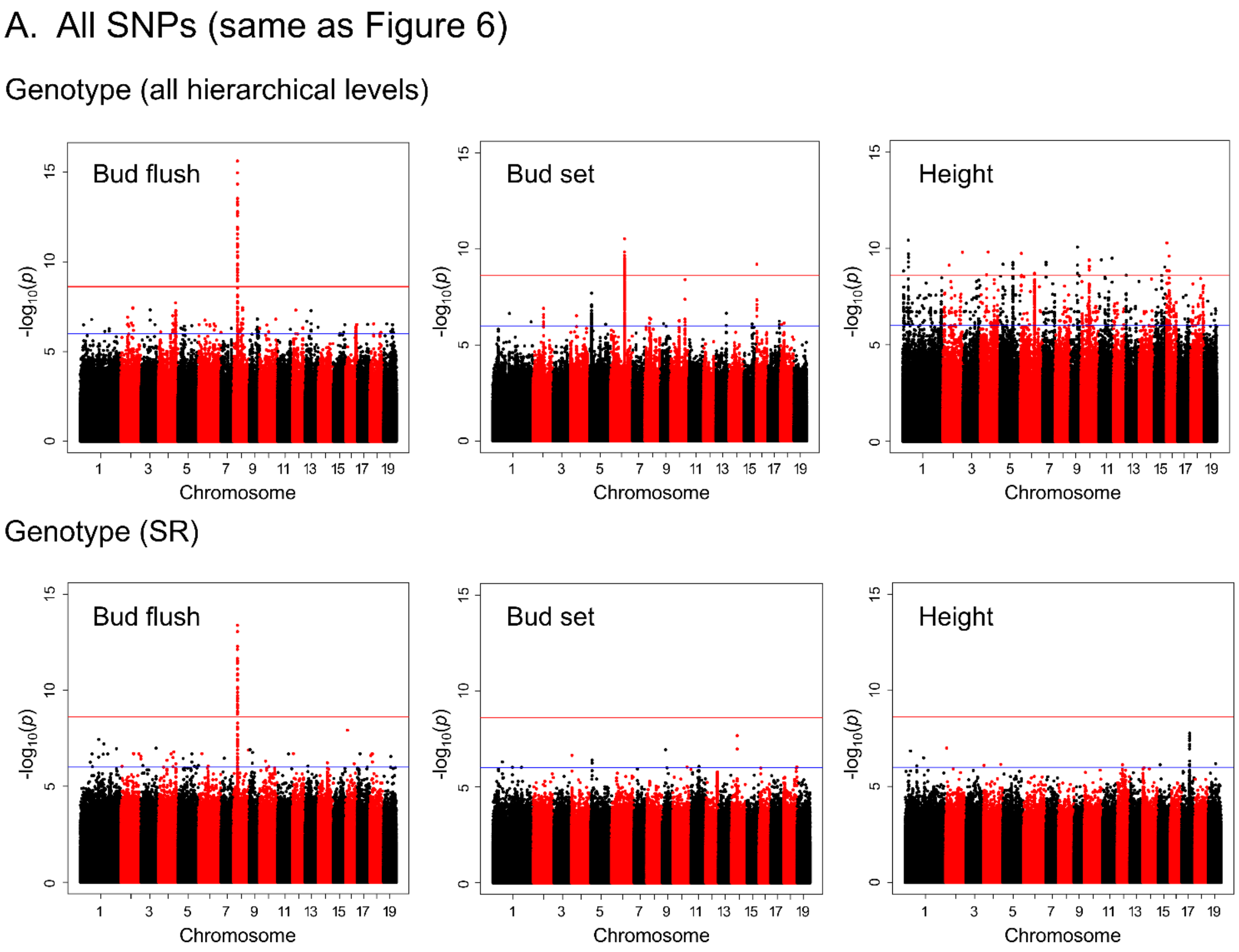

Figure S2. Genome-wide association study (GWAS) results. Results are for vegetative bud flush, bud set, and height based on total genetic variation across rivers, stands, and genotypes (top panels) and among genotypes within stands (bottom panels). (A, C, E, G) Manhattan plots and (B, D, F, H) quantile-quantile (Q-Q) plots from GWAS based on all 20,770,783 SNPs filtered using the 'liberal' criteria (Table S4). (A, B) Same as Fig. 6 in the main text. (C, D) The first five SNP principal components (SPC1-SPC5) were used to correct for population structure. (E, F) Same as for (C, D), except analyses excluded SNPs with MAF < 0.01. (G, H) Same as for (A, B), except analyses were restricted to 490 genotypes from the Skagit, Puyallup, and Columbia Rivers. The blue line on the Manhattan plots indicates a *P*-value of 10^{-6}, and the red line indicates a Bonferroni-corrected *P*-value of 2.4×10^{-9} ($\alpha = 0.05$). The red line on the quantile-quantile (Q-Q) plots indicates the 1:1 diagonal.

B. All SNPs

Genotype (all hierarchical levels)

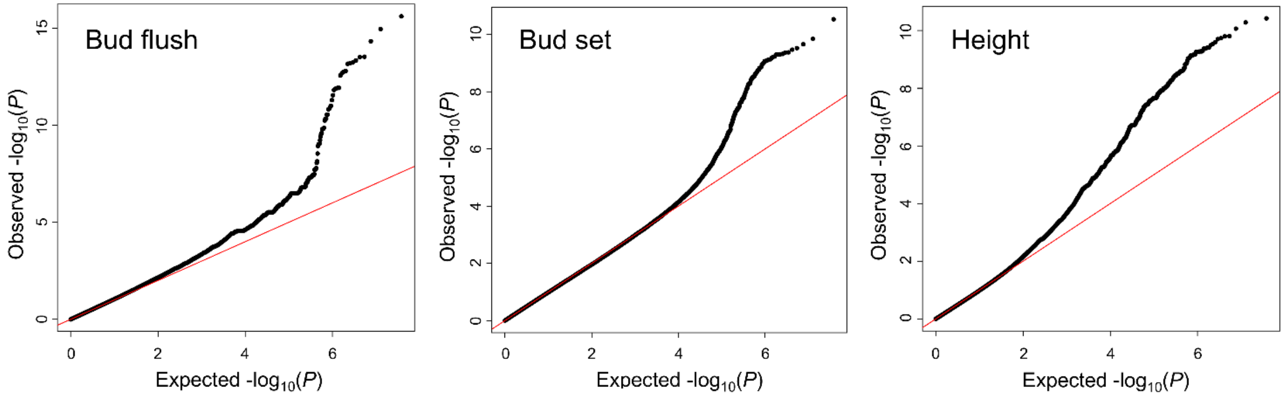

Genotype (SR)

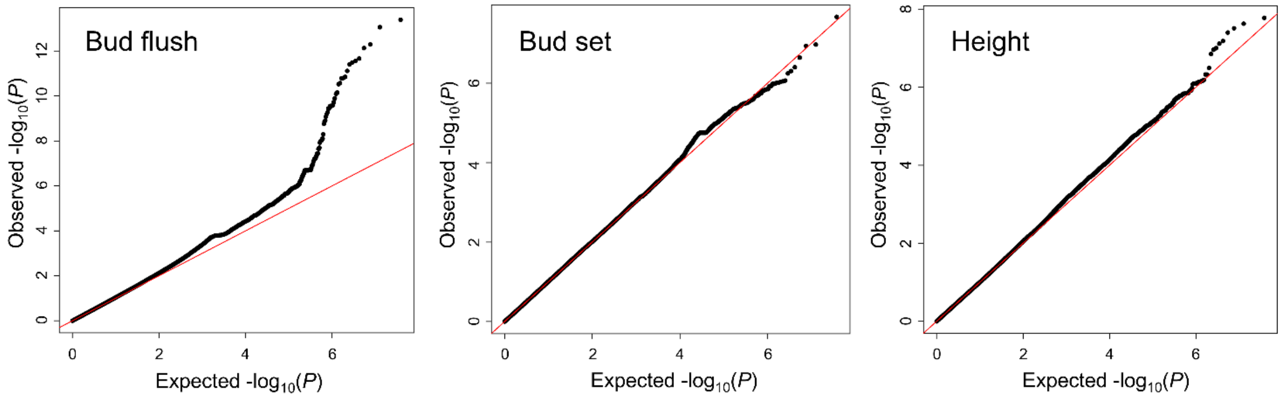

Figure S2 (cont). Genome-wide association study (GWAS) results. Results are for vegetative bud flush, bud set, and height based on total genetic variation across rivers, stands, and genotypes (top panels) and among genotypes within stands (bottom panels). (A, C, E, G) Manhattan plots and (B, D, F, H) quantile-quantile (Q-Q) plots from GWAS based on all 20,770,783 SNPs filtered using the 'liberal' criteria (Table S4). (A, B) Same as Fig. 6 in the main text. (C, D) The first five SNP principal components (SPC1-SPC5) were used to correct for population structure. (E, F) Same as for (C, D), except analyses excluded SNPs with MAF < 0.01. (G, H) Same as for (A, B), except analyses were restricted to 490 genotypes from the Skagit, Puyallup, and Columbia Rivers. The blue line on the Manhattan plots indicates a *P*-value of 10^{-6}, and the red line indicates a Bonferroni-corrected *P*-value of 2.4×10^{-9} ($\alpha = 0.05$). The red line on the quantile-quantile (Q-Q) plots indicates the 1:1 diagonal.

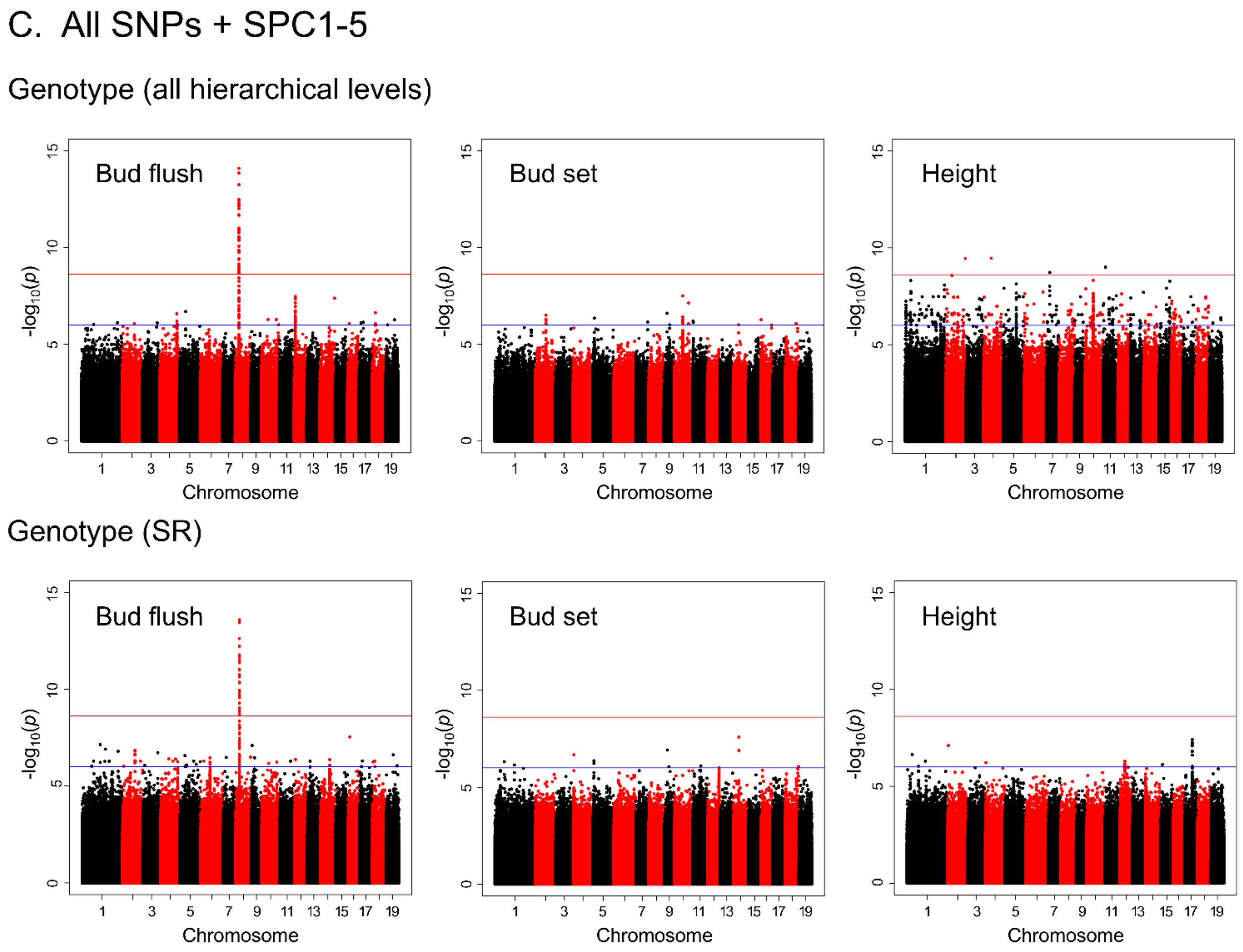

Figure S2 (cont). Genome-wide association study (GWAS) results. Results are for vegetative bud flush, bud set, and height based on total genetic variation across rivers, stands, and genotypes (top panels) and among genotypes within stands (bottom panels). (A, C, E, G) Manhattan plots and (B, D, F, H) quantile-quantile (Q-Q) plots from GWAS based on all 20,770,783 SNPs filtered using the 'liberal' criteria (Table S4). (A, B) Same as Fig. 6 in the main text. (C, D) The first five SNP principal components (SPC1-SPC5) were used to correct for population structure. (E, F) Same as for (C, D), except analyses excluded SNPs with MAF < 0.01. (G, H) Same as for (A, B), except analyses were restricted to 490 genotypes from the Skagit, Puyallup, and Columbia Rivers. The blue line on the Manhattan plots indicates a *P*-value of 10^{-6}, and the red line indicates a Bonferroni-corrected *P*-value of 2.4×10^{-9} ($\alpha = 0.05$). The red line on the quantile-quantile (Q-Q) plots indicates the 1:1 diagonal.

D. All SNPs + SPC1-5

Genotype (all hierarchical levels)

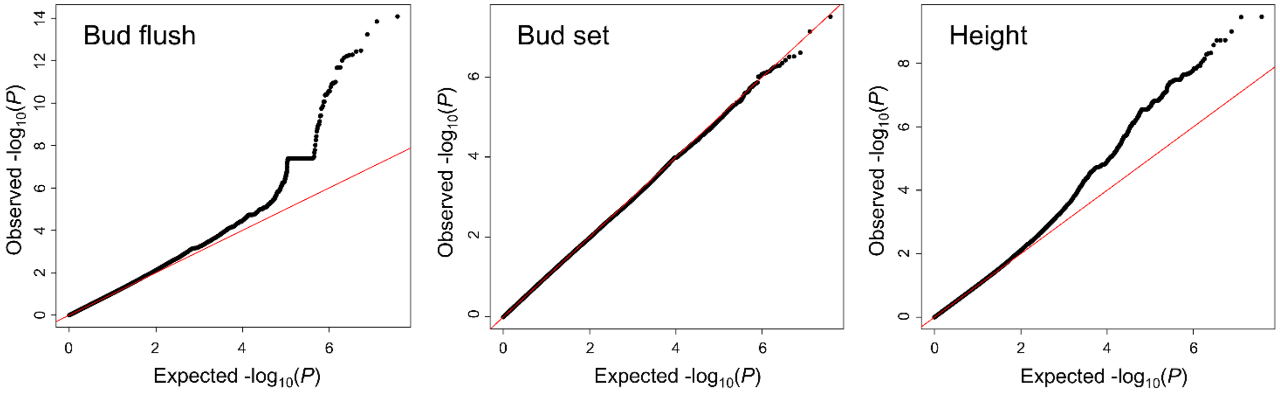

Genotype (SR)

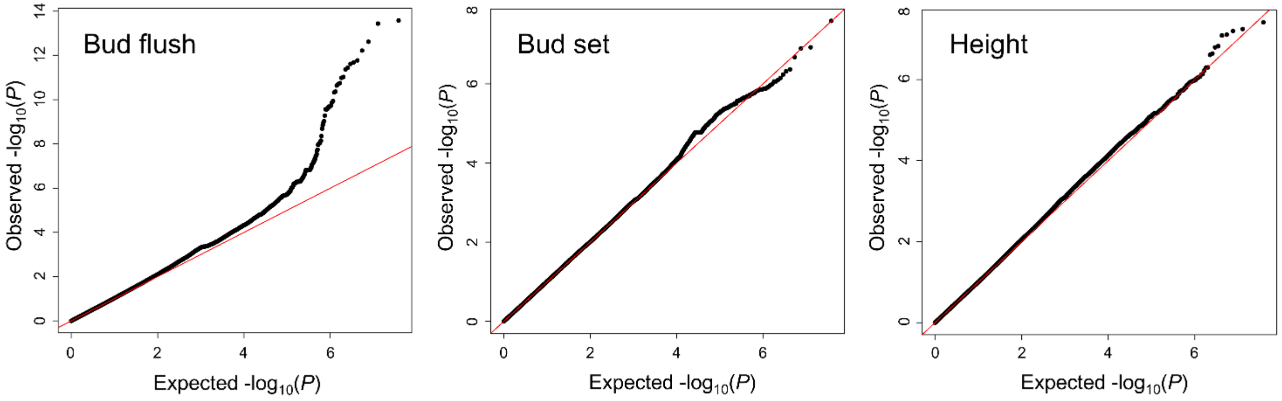

Figure S2 (cont). Genome-wide association study (GWAS) results. Results are for vegetative bud flush, bud set, and height based on total genetic variation across rivers, stands, and genotypes (top panels) and among genotypes within stands (bottom panels). (A, C, E, G) Manhattan plots and (B, D, F, H) quantile-quantile (Q-Q) plots from GWAS based on all 20,770,783 SNPs filtered using the 'liberal' criteria (Table S4). (A, B) Same as Fig. 6 in the main text. (C, D) The first five SNP principal components (SPC1-SPC5) were used to correct for population structure. (E, F) Same as for (C, D), except analyses excluded SNPs with MAF < 0.01. (G, H) Same as for (A, B), except analyses were restricted to 490 genotypes from the Skagit, Puyallup, and Columbia Rivers. The blue line on the Manhattan plots indicates a *P*-value of 10^{-6}, and the red line indicates a Bonferroni-corrected *P*-value of 2.4×10^{-9} ($\alpha = 0.05$). The red line on the quantile-quantile (Q-Q) plots indicates the 1:1 diagonal.

E. SNPs with MAF ≥ 0.01 + SPC1-5

Genotype (all hierarchical levels)

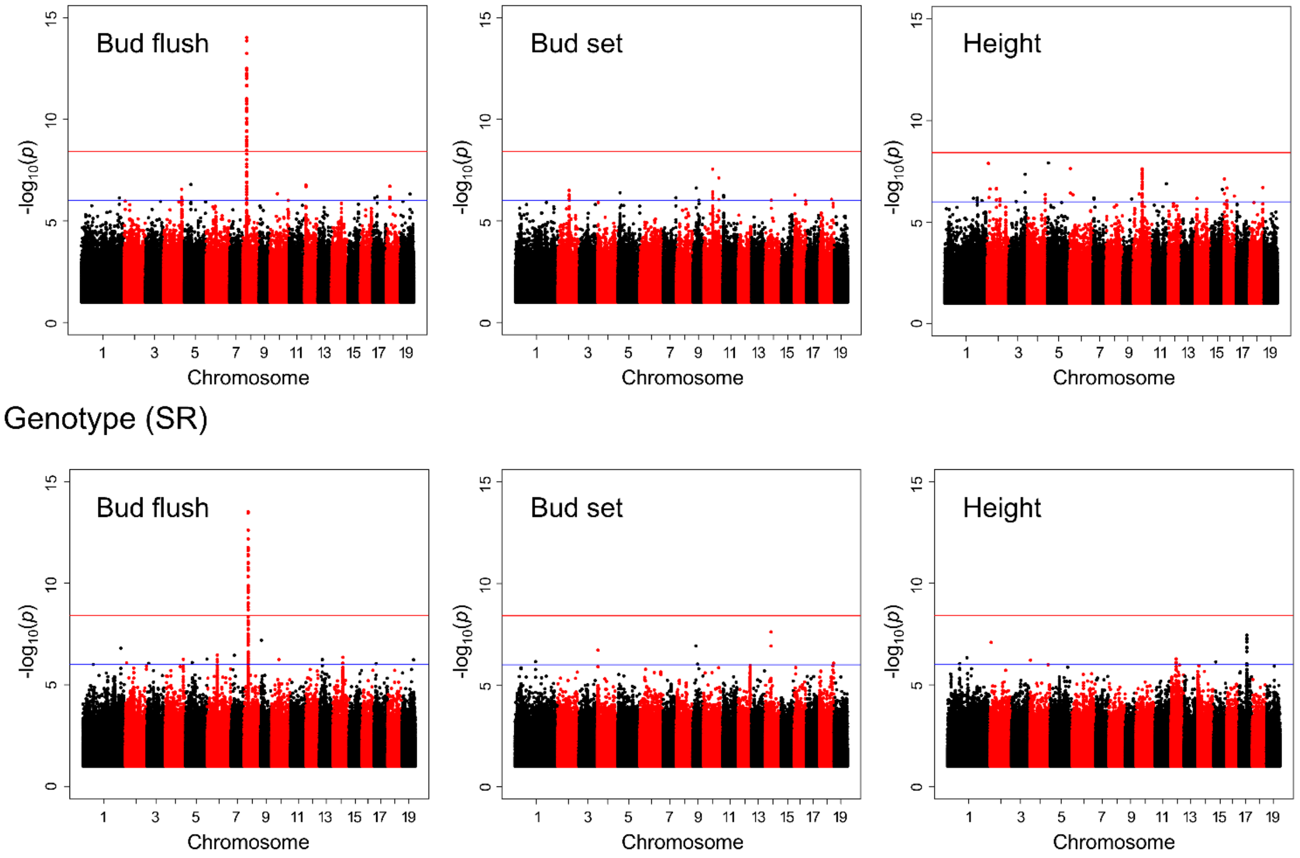

Figure S2 (cont). Genome-wide association study (GWAS) results. Results are for vegetative bud flush, bud set, and height based on total genetic variation across rivers, stands, and genotypes (top panels) and among genotypes within stands (bottom panels). (A, C, E, G) Manhattan plots and (B, D, F, H) quantile-quantile (Q-Q) plots from GWAS based on all 20,770,783 SNPs filtered using the 'liberal' criteria (Table S4). (A, B) Same as Fig. 6 in the main text. (C, D) The first five SNP principal components (SPC1-SPC5) were used to correct for population structure. (E, F) Same as for (C, D), except analyses excluded SNPs with MAF < 0.01. (G, H) Same as for (A, B), except analyses were restricted to 490 genotypes from the Skagit, Puyallup, and Columbia Rivers. The blue line on the Manhattan plots indicates a *P*-value of 10^{-6}, and the red line indicates a Bonferroni-corrected *P*-value of 2.4×10^{-9} ($\alpha = 0.05$). The red line on the quantile-quantile (Q-Q) plots indicates the 1:1 diagonal.

F. SNPs with MAF ≥ 0.01 + SPC1-5

Genotype (all hierarchical levels)

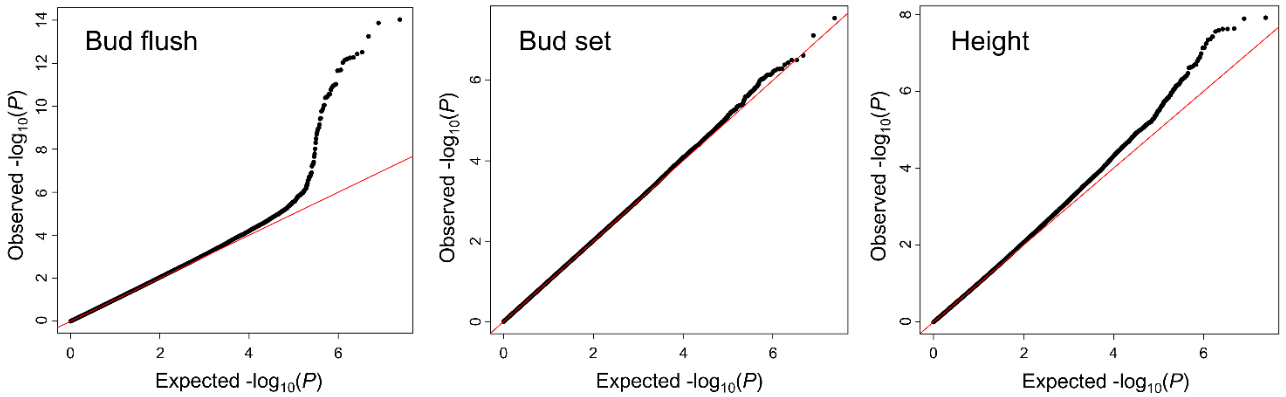

Genotype (SR)

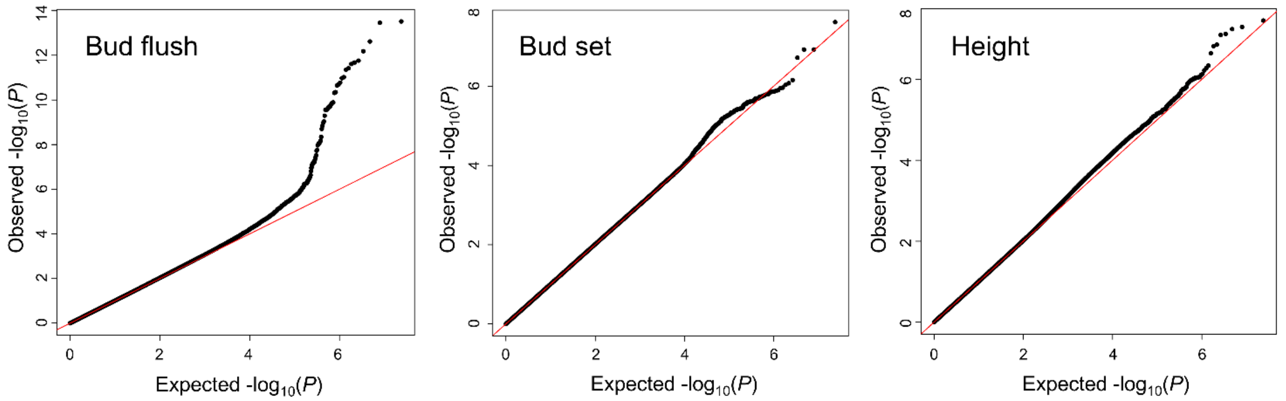

Figure S2 (cont). Genome-wide association study (GWAS) results. Results are for vegetative bud flush, bud set, and height based on total genetic variation across rivers, stands, and genotypes (top panels) and among genotypes within stands (bottom panels). (A, C, E, G) Manhattan plots and (B, D, F, H) quantile-quantile (Q-Q) plots from GWAS based on all 20,770,783 SNPs filtered using the ‘liberal’ criteria (Table S4). (A, B) Same as Fig. 6 in the main text. (C, D) The first five SNP principal components (SPC1-SPC5) were used to correct for population structure. (E, F) Same as for (C, D), except analyses excluded SNPs with MAF < 0.01. (G, H) Same as for (A, B), except analyses were restricted to 490 genotypes from the Skagit, Puyallup, and Columbia Rivers. The blue line on the Manhattan plots indicates a *P*-value of 10^{-6}, and the red line indicates a Bonferroni-corrected *P*-value of 2.4×10^{-9} ($\alpha = 0.05$). The red line on the quantile-quantile (Q-Q) plots indicates the 1:1 diagonal.

G. All SNPs with N = 490 from Skagit, Puyallup, and Columbia

Genotype (all hierarchical levels)

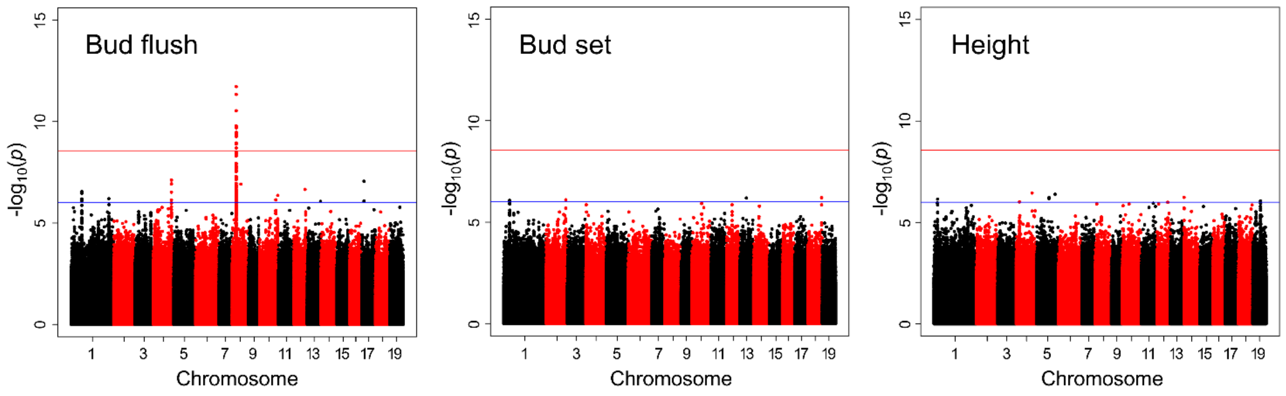

Genotype (SR)

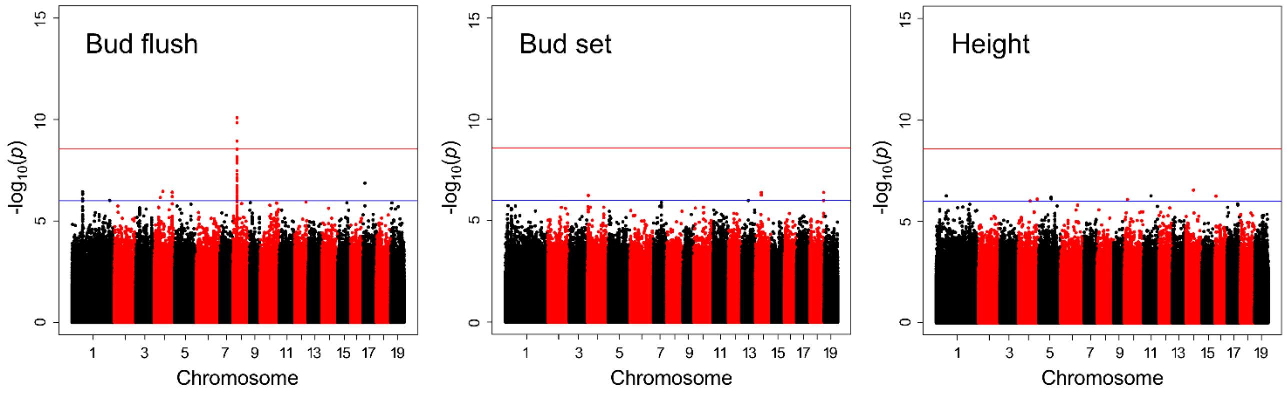

Figure S2 (cont). Genome-wide association study (GWAS) results. Results are for vegetative bud flush, bud set, and height based on total genetic variation across rivers, stands, and genotypes (top panels) and among genotypes within stands (bottom panels). (A, C, E, G) Manhattan plots and (B, D, F, H) quantile-quantile (Q-Q) plots from GWAS based on all 20,770,783 SNPs filtered using the 'liberal' criteria (Table S4). (A, B) Same as Fig. 6 in the main text. (C, D) The first five SNP principal components (SPC1-SPC5) were used to correct for population structure. (E, F) Same as for (C, D), except analyses excluded SNPs with MAF < 0.01. (G, H) Same as for (A, B), except analyses were restricted to 490 genotypes from the Skagit, Puyallup, and Columbia Rivers. The blue line on the Manhattan plots indicates a *P*-value of 10^{-6}, and the red line indicates a Bonferroni-corrected *P*-value of 2.4×10^{-9} ($\alpha = 0.05$). The red line on the quantile-quantile (Q-Q) plots indicates the 1:1 diagonal.

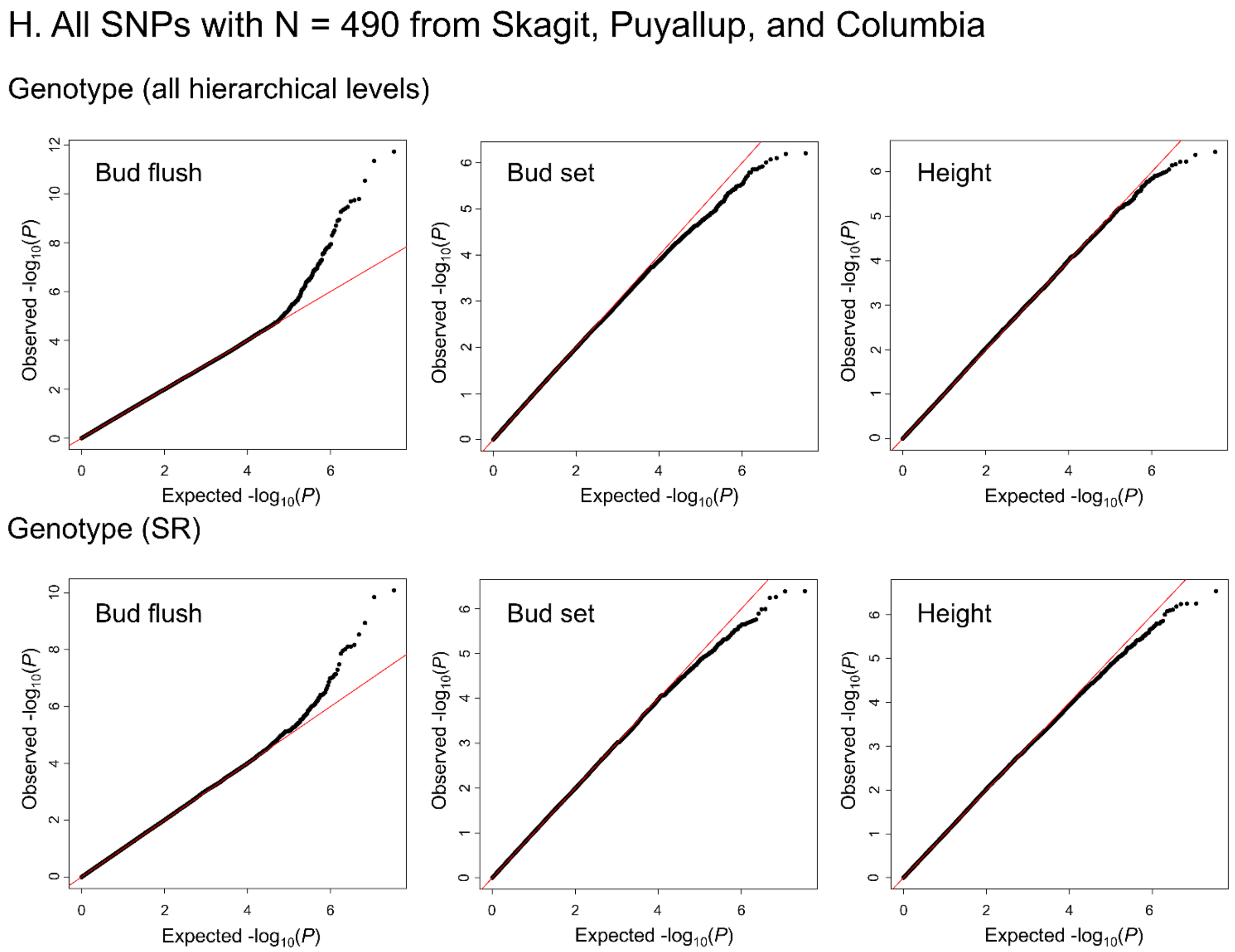

Figure S2 (cont). Genome-wide association study (GWAS) results. Results are for vegetative bud flush, bud set, and height based on total genetic variation across rivers, stands, and genotypes (top panels) and among genotypes within stands (bottom panels). (A, C, E, G) Manhattan plots and (B, D, F, H) quantile-quantile (Q-Q) plots from GWAS based on all 20,770,783 SNPs filtered using the ‘liberal’ criteria (Table S4). (A, B) Same as Fig. 6 in the main text. (C, D) The first five SNP principal components (SPC1-SPC5) were used to correct for population structure. (E, F) Same as for (C, D), except analyses excluded SNPs with MAF < 0.01. (G, H) Same as for (A, B), except analyses were restricted to 490 genotypes from the Skagit, Puyallup, and Columbia Rivers. The blue line on the Manhattan plots indicates a *P*-value of 10^{-6}, and the red line indicates a Bonferroni-corrected *P*-value of 2.4×10^{-9} ($\alpha = 0.05$). The red line on the quantile-quantile (Q-Q) plots indicates the 1:1 diagonal.

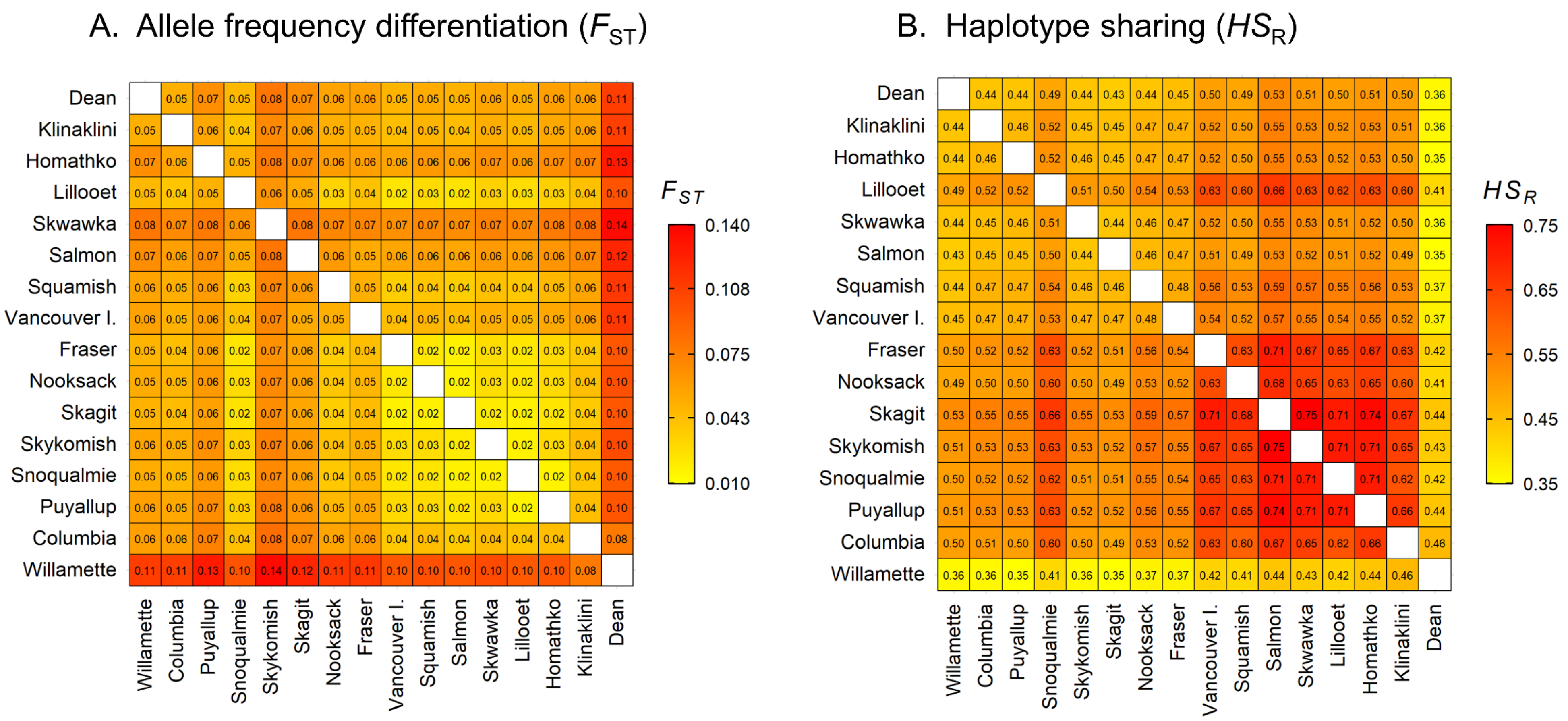

Figure S3. Allele frequency differentiation (F_{ST}) and linkage phase consistency (haplotype sharing, HS_R) among rivers. (A) Pairwise F_{ST} values (Hudson's estimator) were calculated at the river level using SMARTPCA. (B) Haplotype sharing analyses were based on 1,082,633 SNPs with MAF ≥ 0.01 separated by at least 300 bp and haplotype sharing was calculated for all pairs of SNPs located within 10 kb of each other.

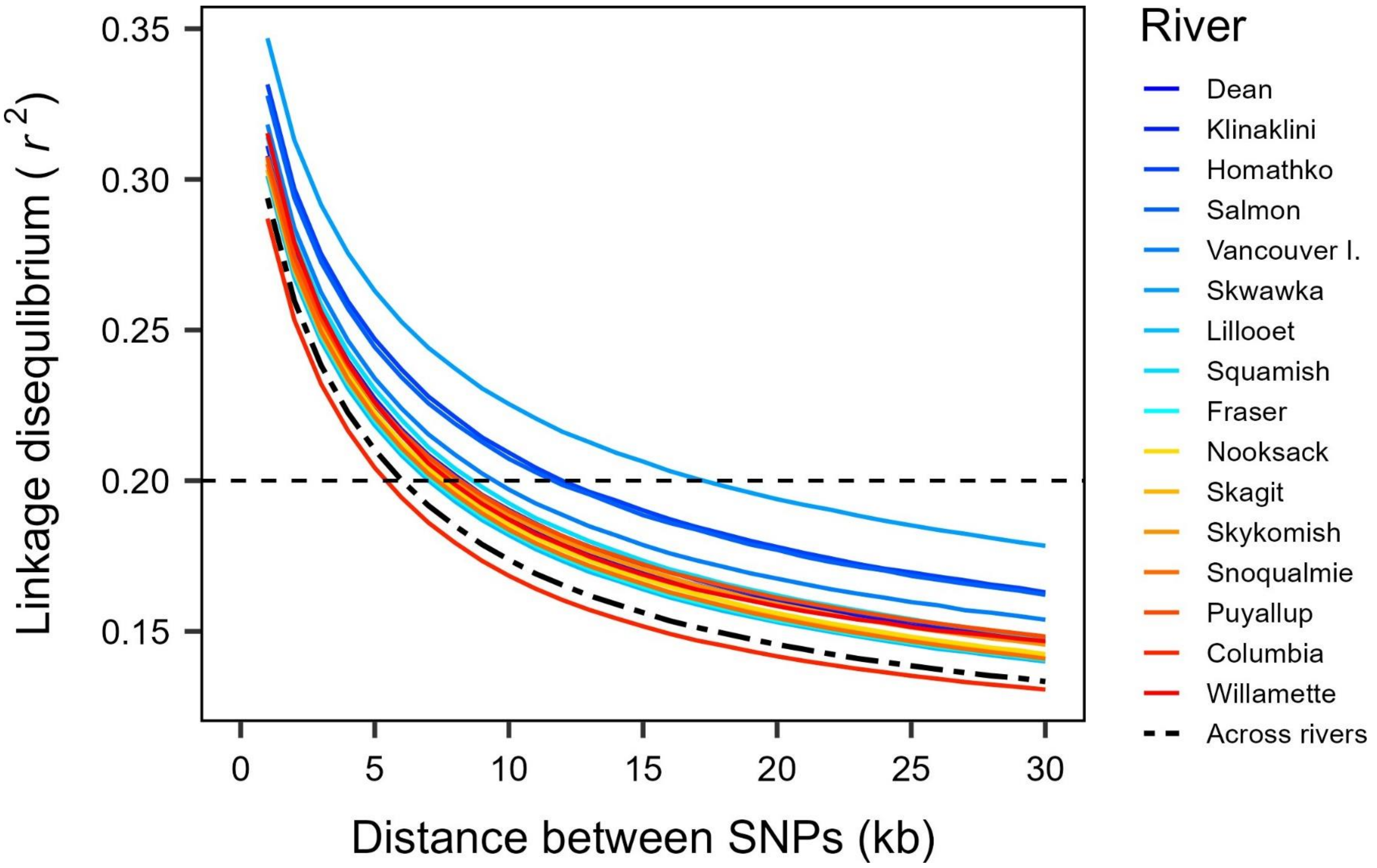

Figure S4. Linkage disequilibrium (LD) within and across rivers. LD was calculated as the average r^2 for pairs of SNPs with MAF ≥ 0.10 in each 1-kb distance class. To equalize sample sizes, N = 10 clonal genotypes were randomly sampled from each river and 10 were sampled across rivers (i.e., one from each of 10 randomly sampled rivers). Average r^2 values were calculated based on 10 random sampling replications and corrected for the small sample size by subtracting $1/2N$. Because of the large number of SNPs, the standard error of each data point was near zero.

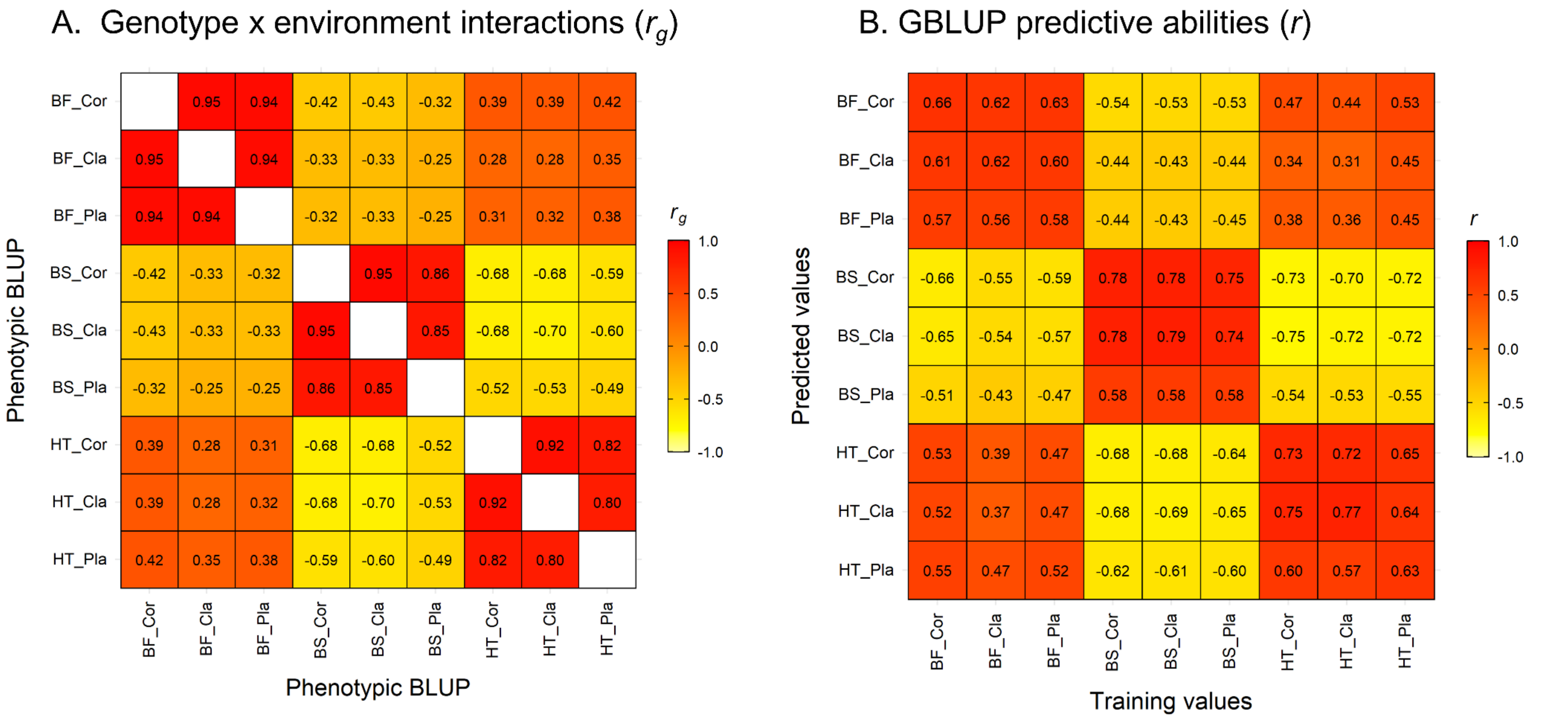

Figure S5. Extent of genotype by environment (GxE) interactions based on among-plantation genetic correlations and predictive abilities (PA). (A) Off-diagonals are the genetic correlations between pairs of plantations for bud flush (BF), bud set (BS), and height growth (HT). (B) GBLUP predictive abilities across all hierarchical levels (genotypes, stands, and rivers) based on 20,770,783 single-nucleotide polymorphism (SNP) markers filtered using the 'liberal' criteria (Table S4). The main diagonal shows the PAs within plantations, whereas the off-diagonals show the PAs across plantations or traits. All predictive abilities are averages from 100 random, ten-fold cross-validations with a training population size of 756 and prediction population size of 84. Plantations are Corvallis (Cor), Clatskanie (Cla), and Placerville (Pla).

A. Vegetative bud flush

Stage	Description	Examples
0	Buds tightly closed	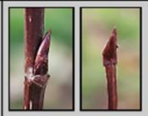
1	Buds swollen/breaking/leaf primordia visible (green visible between scales)	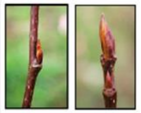
2	Leaves emerging (any leaves showing outside of bud scales)	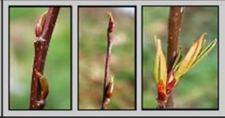
3	Leaves fully emerged and unfolding (leaf petiole is visible)	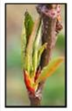
4	Leaves fully unfolded	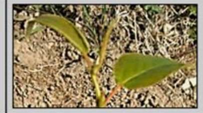
5	Leaves fully expanded	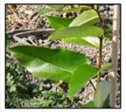

B. Vegetative bud set

Stage	Description	Examples
1	Actively growing<br>Many large new leaves at the apex	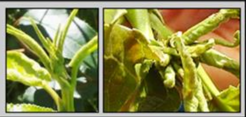
2	Slowing down<br>Some new leaves at the apex	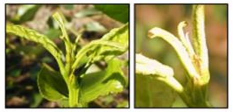
3	Just prior to bud set<br>One small new leaf at the apex<br>Enlarged stipules	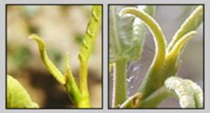
4	Just after bud set<br>Stipules form a point at the apex with no new leaves protruding	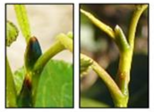
5	Small reddish bud	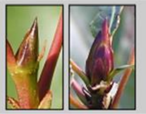
6	Large reddish and woody bud	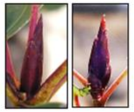

Figure S6. Scores used to quantify the timing of vegetative bud flush and bud set in *Populus trichocarpa*. In this species, the bud scales are formed from modified stipules.

Supporting Materials and Methods

Plant materials and test plantations

We assembled a study population of 1101 black cottonwood clonal genotypes by combining existing and new collections. First, we obtained 282 genotypes collected from western Oregon and western Washington by GreenWood Resources, Inc. (Portland, OR) and 215 genotypes collected from southwestern British Columbia by the British Columbia Ministry of Forests. We also collected new cuttings from large, continuous stands of black cottonwood along four rivers in northwestern Oregon and western Washington. We collected genotypes along the Columbia (N = 140), Puyallup (N = 161), Skykomish (N = 141), and Skagit (N = 140) Rivers, and from five small, isolated stands near Lake Tahoe in California (N = 22). Collections were designed to sample populations that had a common demographic history and were not affected by recent introgression from other *Populus* species (see Figs. 1-1 and 1-2 in DiFazio, Slavov and Joshi (3)). We focused on the portion of the range where black cottonwood has optimal growth, high levels of phenotypic variation, and weak interpopulation differentiation for neutral markers (4-7).

Cuttings collected from the field or arboreta were rooted individually in 115-ml plastic Cone-tainers (SC7, Stuewe & Sons, Inc., Tangent, OR) under greenhouse conditions (Mt. Jefferson Farms, Inc., Salem, OR). Rooted cuttings were overwintered in an outdoor shadehouse at Oregon State University, before outplanting at three sites. The northernmost plantation was at a site owned by GreenWood Resources near Clatskanie, OR. Another plantation was established near Oregon State University in Corvallis. The southernmost test plantation was established at the USDA Forest Service Nursery near Placerville, CA. At each plantation, a single copy of each genotype was planted in each of three randomized complete blocks, and the plantations were fenced to prevent deer and livestock damage. Trees were planted at 3 × 3 m spacing at the Clatskanie and Placerville plantations, and 2 × 3 m at the

Corvallis plantation. Because of the tighter spacing at the Corvallis plantation, it was coppiced after the 2010 growing season. Irrigation was not needed at the Clatskanie plantation, the Corvallis plantation was overhead-irrigated biweekly during the first growing season, and the Placerville plantation (the driest site) was drip-irrigated weekly during each growing season. Plants that died during the first growing season were replanted in the summer of 2010 with filler trees, but these trees were not used in the statistical analyses.

Phenotypic data analysis

Because the blocks were large (i.e., up to ~1100 trees), we accounted for microsite variation using spatial analysis, rather than using a block term in the statistical model. To achieve homogeneity of variance among plantations, we then standardized the raw data at each plantation using the phenotypic standard deviation (8). For single-site analyses, we used these adjusted data and a hierarchical random effects model to calculate variance components and random effects (i.e., best linear unbiased predictors, BLUPs). This model included terms for the plantation mean, river (R), stand-within-river [S(R)], genotype-within-stand-and-river [G(SR)], and error. The across-site model included additional terms for the random effect of plantation and interactions of plantation with R, S(R), and G(SR). These analyses involved five steps. First, for each plantation, we adjusted the raw data using SAS PROC TPSPLINE (SAS v9.4, SAS Institute Inc., Cary, NC). Second, we used a statistical model with genotype as a random effect to estimate phenotypic variance (σ_{P}^2) as $\sigma_{\mathrm{G}}^2 + \sigma_{\varepsilon}^2$, where σ_{G}^2 was the genotype variance component and σ_{ε}^2 was the error variance estimated using SAS PROC GLIMMIX. Third, to achieve homogeneity of variance among plantations, we then standardized the raw data at each plantation using the phenotypic standard deviation (8). Fourth, we adjusted the standardized raw data, using PROC TPSPLINE. Finally, for the single-site analyses, we used these adjusted data and the following statistical model to

calculate variance components and random effects [best linear unbiased predictors (BLUPs)] using PROC GLIMMIX.

$$X_{jklm} = \mu + R_j + S_k(R_j) + G_l(SR_{kj}) + \varepsilon_{jklm}, \quad (1)$$

where X_{jklm} is the observation for the mth tree of the lth genotype from the kth stand within the jth river; μ is the overall mean; R_j is the random effect of the jth river; $S_k(R_j)$ is the random effect of the kth stand within the jth river; $G_l(SR_{kj})$ is the random effect of the lth genotype from the kth stand and jth river; and ε_{jklm} is the experimental error. However, before running the final analysis, we used this same model to identify and remove outliers that had residuals exceeding three standard deviations.

We used the results from Model 1 primarily to evaluate data quality, whereas all remaining analyses were conducted using the results from the following across-site model:

$$X_{ijklm} = \mu + P_i + R_j + S_k(R_j) + G_l(SR_{kj}) + P_i{\cdot}R_j + P_i{\cdot}S_k(R_j) + P_i{\cdot}G_l(SR_{kj}) + \varepsilon_{ijklm}, \quad (2)$$

where X_{ijklm} is the observation for the mth tree of the lth genotype from the kth stand within the jth river at the ith plantation; P_i is the random effect of the ith plantation; $P_i{\cdot}R_j$, $P_i{\cdot}S_k(R_j)$, and $P_i{\cdot}G_l(SR_{kj})$ are the interactions between the ith plantation and the jth river, kth stand within the jth river, and the lth genotype from the kth stand within the jth river; and other effects are as described above.

For each *P. trichocarpa* genotype, we calculated two ‘phenotypes,’ G(SR) and G, by combining random effects from Model 2. G(SR) includes only the G(SR) random effect, whereas G includes the random effects from all three hierarchical levels, G(SR), S(R), and R. We then estimated genetic correlations among traits (within and among plantations) as the Pearson product-moment correlations among these values.

We used the variance components from Model 2 to calculate heritabilities and characterize the distribution of genetic variation at different spatial scales. We estimated variance components for rivers (σ^2_{R}), stands-within-rivers ($\sigma^2_{\mathrm{S(R)}}$), genotypes-within-stands-

and-rivers ($\sigma^2_{\mathrm{G(SR)}}$), and error ($\sigma^2_{\varepsilon}$). $\sigma^2_{\mathrm{G(SR)}}$ represents within-stand genetic variation, whereas the sum of σ^2_{R}, $\sigma^2_{\mathrm{S(R)}}$, and $\sigma^2_{\mathrm{G(SR)}}$ is the total genetic variation (σ^2_{G}). Additionally, we estimated variance components for the plantation (environment or E) by genetic interactions, $\sigma^2_{\mathrm{R \cdot E}}$, $\sigma^2_{\mathrm{S(R) \cdot E}}$, and $\sigma^2_{\mathrm{G(SR) \cdot E}}$, the sum of which is the total genotype-by-environmental (GxE) variance ($\sigma^2_{\mathrm{G \cdot E}}$).

We calculated total heritabilities as $H^2_G = \sigma^2_{\mathrm{G}}/(\sigma^2_{\mathrm{G}} + \sigma^2_{\mathrm{G \cdot E}} + \sigma^2_{\varepsilon})$, and within-stand heritabilities as $H^2_{G(SR)} = \sigma^2_{\mathrm{G(SR)}}/(\sigma^2_{\mathrm{G(SR)}} + \sigma^2_{\mathrm{G(SR) \cdot E}} + \sigma^2_{\varepsilon})$. Proportions of genotypic variation were calculated as $\sigma^2_{\mathrm{R}}/\sigma^2_{\mathrm{G}}$ for rivers, $\sigma^2_{\mathrm{S(R)}}/\sigma^2_{\mathrm{G}}$ for stands-within-rivers, and $\sigma^2_{\mathrm{G(SR)}}/\sigma^2_{\mathrm{G}}$ for genotypes-within-stands. To further characterize genetic structure, we calculated among-stand Q_{ST} values at each hierarchical level. Q_{ST}, a measure of allelic differentiation for quantitative traits, was calculated as follows (9, 10): $Q_{\mathrm{ST}} = (\sigma^2_{\mathrm{R}} + \sigma^2_{\mathrm{S(R)}})/(\sigma^2_{\mathrm{R}} + \sigma^2_{\mathrm{S(R)}} + 2\sigma^2_{\mathrm{G(SR)}})$. Finally, to illustrate spatial patterns of genetic structure, we performed principal component analysis (PCA) of the data matrix consisting of G values for the three phenotypic traits in each of the three test plantations (i.e., nine variables). This was done using the *prcomp* function in R (11), with the *center* and *scale* options set to TRUE.

SNP data

Although we measured phenotypes for 1101 trees, only 970 trees were sequenced to obtain SNP data. To remove related trees, we clustered the 970 sequenced trees into seven groups using fastSTRUCTURE (12). Next, we calculated a genomic relationship matrix (GRM) for each group using the *--make-grm-gz* option of GCTA (13). Finally, we removed one individual from each pair that had a GRM value exceeding 0.25. We excluded 42 other individuals for four reasons. We removed 12 individuals because they could not be grouped into stands and rivers with the other clonal genotypes (note, 'river' denotes a river or

drainage). Second, we removed all 13 individuals from an atypical, strongly differentiated subpopulation from Lake Tahoe (pairwise $F_{ST} > 0.15$) (6, 14). Third, we removed all individuals from three rivers that had < 10 individuals each (Kitimat, Nisqually, and Cowlitz), resulting in the removal of another 14 individuals. Finally, we removed three individuals that did not cluster within their respective rivers based on the first two SNP principal components (SPC1 and SPC2) (i.e., were > 3 standard deviations from the river centroids). The remaining 840 clonal genotypes represented 91 stands in 16 rivers (main text Fig. 1).

For the final analyses, we filtered SNPs based on three sets of criteria (Table S4) using VCFtools v.0.1.14 (15) and PLINK v.1.90b4.4 (16). We used 'strict' filtering to set SNP genotypes (AA, AB, BB) with < 8 reads or genotype quality < 20 as missing data (*vcftools --minDP* 8 *--minGQ* 20), and then excluded SNPs with minor allele frequency (MAF) < 0.01 (*vcftools --maf* 0.01) or > 10% missing data (*plink --geno* 0.1). For maximum genome coverage, we used 'liberal' filtering to retain all SNPs with at least 2 copies of the minor allele (*vcftools --mac* 2), and did not filter based on the number of reads per genotype, genotype quality, proportion of missing data, or MAF. Finally, we also simulated a set of 51,820 'RAD-Seq' SNPs based on high-quality RAD loci that were detected in each of seven *P. alba* and seven *P. tremula* genotypes using *PstI* and a minimum sequencing coverage of 6× (17). We translated the positions of these known loci from version 2 to version 3 of the *P. trichocarpa* genome (18), and then selected all bi-allelic, non-singleton SNPs from our re-sequencing data that were within 100-bp of a *PstI* restriction site. Because *PstI* is methylation sensitive, some loci are not 'RAD-accessible,' and will be missed. Thus, this approach masks our re-sequencing data to simulate the RAD-accessible portion of the genome.

GBLUP

We tested the effect of training population sizes (N_t) ranging from 48 to 756, while keeping the prediction population size (N_p) fixed at 84. This was done by randomly sampling $N_t + N_p$ clonal genotypes, and then randomly allocating them to the training versus prediction population. Similarly, for river-specific GBLUP models, we varied N_t between 24 and 96, while N_p was fixed at 64. For all other GBLUP analyses, we used random, ten-fold cross-validation ($N_t = 756$, $N_p = 84$). To assess the effect of the number of markers used (M), we randomly selected $10^1 – 10^7$ SNPs using PLINK (*plink --thin-count*). The performance of GBLUP was measured by predictive ability (PA), which are the Pearson product-moment correlation coefficients between the input phenotypes and the phenotypes predicted from the SNP data (19, 20). For testing the effect of N_t, we calculated PAs based on the 64 or 84 predicted values (N_p) described above, but for the other analyses using 10-fold cross-validation, PAs were based on all 840 predicted values. We calculated PAs at the river and stand-within-river levels by calculating the correlation between the R and S(R) phenotypes versus the average predicted values from GBLUP for the clonal genotypes in each river or stand. Standard errors of PA means were calculated as $[var(r)/k + (1 - r^2)^2/(N - 2)]^{1/2}$, where the first term is the empirical variance of the mean PA across k replications and the second term is the sampling variance of a Pearson correlation coefficient for $N = N_t + N_p$ genotypes. Finally, we compared the GBLUP approach described above (i.e., based on the GRM alone) to models that also included the first five PC scores of the genomic relationship matrix as fixed-effect covariates (21).

Linkage disequilibrium and haplotype sharing

We calculated r^2 for each pair of SNPs located within 10 kb of each other using PLINK (*plink --r2 --ld-window 1000 --ld-window-kb 10 --ld-window-r2 0*) using different MAF cut-

offs or bins. The MAF bins (Table S5) were set so that all pairs of loci in a bin had potentially high LD [i.e., an r^2 with a theoretical maximum of at least 0.5 (2)]. To estimate the probability of tagging a QTN, we first selected $M = 10^2 – 10^7$ SNPs at random using PLINK (*plink --thin-count*), then randomly selected a putative QTN from the *M* SNPs and calculated r^2 between the QTN and all SNPs located within 10 kb from it. This was repeated 100 times for each value of *M* and the estimate of tagging probability was calculated as the proportion of times at least one SNP within 10 kb had $r^2 \geq 0.6$ with the putative QTN.

To compare LD within and among rivers, we randomly sampled 10 clonal genotypes from each river and 1 genotype from each of 10 randomly sampled rivers. This approach equalized the within-river and among-river comparisons (i.e., the minimum sample size within river was $N = 10$), which is important because r^2 is sensitive to sample size (22). Next, we calculated pairwise r^2 as described above using 10 replications. For these analyses, we eliminated singleton SNPs from each sample (MAF ≥ 0.10), calculated r^2 only for SNPs that were separated by at least 300 bp, and then corrected r^2 for the small sample size by subtracting $1/2N$.

To quantify linkage phase consistency, we first selected a subset of SNPs with MAF ≥ 0.01 that were separated by at least 300 bp and located on chromosomes 1-19. We then calculated the genotypic correlations (g_r) between all pairs of SNPs from that set that were located within 10 kb of each other using PLINK (*plink --r --ld-window 50 --ld-window-kb 10*). We used haplotype sharing (*HS*) as our measure of linkage phase consistency (23-25). To calculate haplotype sharing among rivers (HS_R), we calculated g_r values for each locus pair and river, calculated the correlation between g_r values for each pair of rivers (r_{gr}), and then calculated HS_R as the overall average of all r_{gr} values across all pairs of rivers. We used an analogous approach to calculate haplotype sharing among stands-in-rivers ($HS_{S(R)}$), but only for the Columbia, Skykomish, and Skagit rivers. These were the only rivers that had

multiple stands with at least 20 clones each. Finally, we calculated haplotype sharing among genotypes-within-stands-and-rivers ($HS_{G(SR)}$) by sampling four stands (each in a different river) that had at least 40 clones and randomly splitting clones in each stand into two groups of at least 20 clones to calculate within stand r_{gr}. To minimize the effects of allele frequency differentiation on haplotype sharing, we also repeated this analysis for a set of SNPs with $0.01 \leq$ MAF < 0.11 in each of the 16 rivers. Because very few SNPs satisfied these criteria (M = 1002), we extended the window in which we calculated haplotype sharing to 1 Mb. Standard errors of haplotype sharing (HS) means were calculated as $[var(HS)/k]^{1/2}$, where k is the number of pairs of rivers, stands or samples of genotypes within stands for which HS values were calculated.

Random forest and ridge regression analyses

We used random forest regression and ridge regression to compare the abilities of climatic and geographic variables to predict quantitative traits and SNP PC scores at the stand (S) and river (R) levels. For each approach, we calculated predictive abilities for four sets of predictor variables: (1) geographic variables (latitude, longitude, and elevation), (2) 21 temperature and precipitation related variables from ClimateNA v5.21 [1961-1990 normals (1)], (3) a subset of climate variables selected using random forest regression at the stand level, and (4) all geographic and climatic variables.

To select the most important climate variables (i.e., climate variable subset), we first used random forest regression at the stand level. These analyses resulted in a separate set of selected climate variables for each phenotypic trait and SNP PC score (Tables S1 and S2). Next, we used these selected climate variables and the other predictors described above to calculate PA as the correlation between the phenotypic BLUP or SNP PC score versus the predictions from the regression models. After the independent variables were centered and

scaled using the R *scale* function, both linear and squared values were included in the models. Random forest regression was conducted using the *randomForest* function from the *randomForest* R package v. 4.6.14 (26) using default parameters, ntree = 2000, sampsize = 2/3, and replace = FALSE. Ridge regression was conducted using the *cv.glmnet* function from the *glmnet* R package (27) using default parameters, s = lambda.min and alpha = 0 (ridge regression). These functions were run using a random cross-validation approach with 8 folds at the river level (N = 16) and 10 folds at the stand level (N = 91). Each cross-validation analysis was run 100 times and the mean correlation (PA) and its standard error were calculated.

*Delineation of seed zones*We defined three types of 'true' seed zones: 16 seed zones corresponding to the 16 rivers, 16 seed zones delineated by clustering phenotypes, and 3 seed zones delineated by clustering phenotypes. We then used cluster purity (CP) to measure the success of reconstructing the true seed zones using phenotypes (river seed zones only), SNPs, climate variables, or geographic variables. Cluster purity is the proportion of stands that were both in the same true seed zone and in the same reconstructed seed zone (28). Zones were delineated and reconstructed by clustering the stand-level PC scores of phenotypes, SNPs, climate variables, and geographic variables using the R *kmeans* function, with the *centers* option set to the number of desired zones and *nstart* set to 25. The phenotypic data consisted of stand-level averages for the first 5 PCs derived from a PCA of 9 phenotypes (i.e., 3 traits measured in 3 plantations). SNP data consisted of stand-level averages for the first 5 PCs derived from a PCA of the GRM calculated using SNPs filtered with the 'liberal' criteria (Table S4). Climate and geographic data consisted of stand-level averages for the first 5 PCs (climate) or 3 PCs (geography) derived from PCAs of the same variables used for the random forest and ridge regression analyses described above (*SI Dataset* S1). Standard errors of CP

means were calculated as $[var(CP)/k + CP(1 - CP)/N]^{1/2}$, where the first term is the empirical variance of the mean CP across *k* replications and the second term is the sampling variance of CP (a proportion) with *N* = 91 stands.

SI References

1. T. Wang, A. Hamann, D. Spittlehouse, C. Carroll, Locally downscaled and spatially customizable climate data for historical and future periods for North America. *PLOS ONE* **11**, e0156720 (2016).
2. N. R. Wray, Allele frequencies and the r^2 measure of linkage disequilibrium: Impact on design and interpretation of association studies. *Twin Res Hum Genet* **8**, 87-94 (2005).
3. S. P. DiFazio, G. T. Slavov, C. P. Joshi, "*Populus*: A premier pioneer system for plant genomics" in Genetics, Genomics and Breeding of Poplar, C. P. Joshi, S. P. DiFazio, C. Kole, Eds. (Science Publishers, Enfield, NH, 2011), pp. 1-28.
4. J. C. Weber, R. F. Stettler, Isoenzyme variation among ten populations of *Populus trichocarpa* (Torr. & Gray) in the Pacific Northwest. *Silvae Genet* **30**, 82-87 (1981).
5. J. C. Weber, R. F. Stettler, P. E. Heilman, Genetic variation and productivity of *Populus trichocarpa* and its hybrids. I. Morphology and phenology of 50 native clones. *Can J For Res* **15**, 376-383 (1985).
6. G. T. Slavov *et al.*, Genome resequencing reveals multiscale geographic structure and extensive linkage disequilibrium in the forest tree *Populus trichocarpa*. *New Phytol* **196**, 713-725 (2012).
7. D. S. DeBell, "*Populus trichocarpa* Torr. & Gray, Black Cottonwood" in Silvics of North America Vol. 2. Hardwoods. Agriculture Handbook 654, R. M. Burns, B. H. Honkala, Eds. (U.S. Department of Agriculture, Forest Service, Washington D.C, 1990), pp. 570-576.
8. T. White, "Genetic parameter estimates and breeding value predictions: Issues and implications in tree improvement programs" in *Tree improvement for sustainable tropical forestry QFRI IUFRO Conference*, eds M. J. Dieters, A. C. Matheson, D. G. Nikles, C. E. Harwood, S. M. Walker (Caloundra, Queensland, Australia, 1996), pp 110-117.
9. M. C. Whitlock, Evolutionary inference from Q_{ST}. *Mol Ecol* **17**, 1885-1896 (2008).
10. M. C. Whitlock, K. J. Gilbert, Q_{ST} in a hierarchically structured population. *Mol Ecol Res* **12**, 481-483 (2012).
11. R Core Team, A language and environment for statistical computing. (R Foundation for Statistical Computing, URL http://www.R-project.org/, Vienna, Austria, 2021).
12. A. Raj, M. Stephens, J. K. Pritchard, fastSTRUCTURE: Variational inference of population structure in large SNP data sets. *Genetics* **197**, 573 (2014).
13. J. Yang, S. H. Lee, M. E. Goddard, P. M. Visscher, GCTA: A tool for genome-wide complex trait analysis. *Am J Hum Genet* **88**, 76-82 (2011).
14. L. M. Evans *et al.*, Population genomics of *Populus trichocarpa* identifies signatures of selection and adaptive trait associations. *Nat Genet* **46**, 1089-1096 (2014).
15. P. Danecek *et al.*, The variant call format and VCFtools. *Bioinformatics* **27**, 2156-2158 (2011).
16. C. C. Chang *et al.*, Second-generation PLINK: Rising to the challenge of larger and richer datasets. *Gigascience* **4**, 7 (2015).

17. K. N. Stoelting *et al.*, Genomic scan for single nucleotide polymorphisms reveals patterns of divergence and gene flow between ecologically divergent species. *Mol Ecol* **22**, 842-855 (2013).
18. G. A. Tuskan *et al.*, The genome of black cottonwood, *Populus trichocarpa* (Torr. & Gray). *Science* **313**, 1596-1604 (2006).
19. H. D. Daetwyler, M. P. L. Calus, R. Pong-Wong, G. de Los Campos, J. M. Hickey, Genomic prediction in animals and plants: Simulation of data, validation, reporting, and benchmarking. *Genetics* **193**, 347-365 (2013).
20. G. T. Slavov *et al.*, Genome-wide association studies and prediction of 17 traits related to phenology, biomass and cell wall composition in the energy grass *Miscanthus sinensis*. *New Phytol* **201**, 1227-1239 (2014).
21. C. Y. Chen, J. Han, D. J. Hunter, P. Kraft, A. L. Price, Explicit modeling of ancestry improves polygenic risk scores and BLUP prediction. *Genet Epidemiol* **39**, 427-438 (2015).
22. A. Tenesa *et al.*, Recent human effective population size estimated from linkage disequilibrium. *Genome Res* **17**, 520-526 (2007).
23. A. P. de Roos, B. J. Hayes, R. J. Spelman, M. E. Goddard, Linkage disequilibrium and persistence of phase in Holstein-Friesian, Jersey and Angus cattle. *Genetics* **179**, 1503-1512 (2008).
24. R. A. Gibbs *et al.*, Genome-wide survey of SNP variation uncovers the genetic structure of cattle breeds. *Science* **324**, 528-532 (2009).
25. J. W. Kijas *et al.*, Genome-wide analysis of the world's sheep breeds reveals high levels of historic mixture and strong recent selection. *PLOS Biology* **10**, e1001258 (2012).
26. A. Liaw, M. Wiener, Classification and regression by randomForest, in *R News*, *https://CRAN.R-project.org/doc/Rnews/*, 2002, pp 18-22.
27. J. Friedman, T. Hastie, R. Tibshirani, Regularization paths for generalized linear models via coordinate descent. *J Stat Softw* **33**, 1-22 (2010).
28. D. C. Manning, P. Raghavan, H. Schütze, *Introduction to information retrieval* (Cambridge University Press, Cambridge, UK, 2008), vol. 13.
